# The PKA/MBD2 Axis Transcriptionally Represses INPP5A to Modulate PI3K/Akt Signaling and Accelerate Pituitary Tumorigenesis

**DOI:** 10.1002/cns.70817

**Published:** 2026-03-19

**Authors:** Qian Jiang, Yaorui Wang, Zihan Wang, Quanji Wang, Sihan Li, Linpeng Xu, Zhuo Zhang, Zhoubin Tan, Huaqiu Zhang, Kai Shu, Ting Lei, Yimin Huang, Zhuowei Lei

**Affiliations:** ^1^ Department of Neurosurgery Tongji Hospital of Tongji Medical College of Huazhong University of Science and Technology Wuhan Hubei province China; ^2^ Sino‐German Neuro‐Oncology Molecular Laboratory, Department of Neurosurgery Tongji Hospital of Tongji Medical College of Huazhong University of Science and Technology Wuhan China; ^3^ Department of Orthopedics Tongji Hospital of Tongji Medical College of Huazhong University of Science and Technology Wuhan China

**Keywords:** 14–3‐3σ, INPP5A, MBD2, PitNET, PKA

## Abstract

**Context and Objective:**

The malignant progression of pituitary neuroendocrine tumors (PitNETs) is closely associated with abnormalities in the phosphoinositide signaling pathway. This study aims to investigate the regulatory role and molecular mechanism of inositol polyphosphate 5‐phosphatase A (INPP5A) in the malignant progression of PitNETs, with a focus on its interaction with the PI3K/Akt signaling pathway and the epigenetic regulator MBD2.

**Setting:**

Tongji Hospital of Tongji medical college of Huazhong University of Science and Technology.

**Design:**

Analyze genes related to IP3 metabolism in single‐cell sequencing samples of PitNETs from NCBI, and perform immunofluorescence staining and statistical analysis on samples from 62 patients with PitNETs.

**Result:**

INPP5A was significantly downregulated in PitNETs, and its expression was negatively correlated with tumor invasiveness, Ki67 index, and volume, Overexpression of INPP5A inhibited tumor cell proliferation, migration, and hormone secretion, while knockdown of INPP5A promoted these malignant phenotypes, INPP5A negatively regulated the PI3K/Akt pathway by degrading IP3, MBD2 directly bound to the INPP5A promoter region to mediate transcriptional repression, Activation of PKA signaling phosphorylated MBD2 (at S99), recruited 14‐3‐3σ to stabilize the MBD2 protein, and enhanced the inhibition of INPP5A.

**Conclusion:**

INPP5A acts as a tumor suppressor gene in PitNETs, and its downregulation promotes tumor malignant progression by activating the PI3K/Akt pathway. MBD2 and its PKA‐mediated phosphorylation are key mechanisms for INPP5A transcriptional repression. Targeting the MBD2‐INPP5A‐PI3K/Akt axis may provide a new strategy for the treatment of PitNETs.

## Graphical abstract

Loss of INPP5A in PitNETs disrupts IP3 clearance and drives aberrant PI3K/Akt activation. PKA‐dependent phosphorylation stabilizes MBD2 via 14‐3‐3σ, sustaining transcriptional silencing of INPP5A. MBD2 inhibition sensitizes tumors to standard treatments and limits PitNET growth.

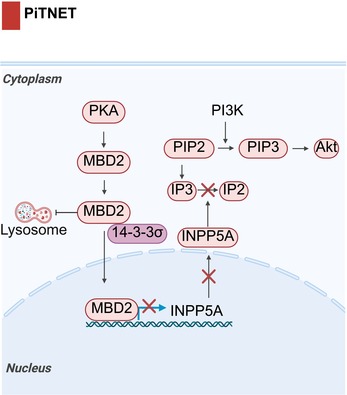

## 1 Introduction

Pituitary neuroendocrine tumor (PitNET), as common tumors of the central nervous system, still pose severe challenges to clinical management due to their invasive growth, recurrence, and abnormal hormone secretion [1, 2]. Despite continuous advancements in surgical and pharmaceutical interventions, there remains an urgent need to understand the core molecular events that drive their malignant progression in order to develop new targeted therapies.

Abnormal activation of the phosphatidylinositol signaling pathway (such as the homeostasis imbalance of the second messenger inositol 1,4,5‐trisphosphate [IP3]) has been confirmed to be closely related to proliferation, invasion and anti‐apoptosis in various tumors. The metabolism of IP3 is critically dependent on inositol polyphosphate 5‐phosphatase A (INPP5A), and the loss of its expression or functional defects may lead to the pathological accumulation of IP3 and excessive activation of downstream oncogenic signals [3, 4, 5]. However, the regulatory mechanism and functional significance of INPP5A in PitNET, especially how upstream signals integrate epigenetic regulation to control its expression, remain important scientific issues that are yet to be resolved.

Methyl‐CpG binding domain protein 2 (MBD2) is an important epigenetic regulator that represses target gene transcription by recognizing methylated DNA in the promoter region and recruiting co‐repressor complexes [6, 7]. However, its specific mechanism of action in PitNET biology remains unclear. Our study found that INPP5A is transcriptionally repressed by MBD2 through binding to the promoter region of INPP5A, thereby promoting the progression of PitNET.

Dysregulation of the protein kinase A (PKA) pathway has been widely reported to be associated with the development of PitNET [8]. However, the specific molecular mechanisms by which PKA downstream regulates the malignant phenotype of tumor cells have not been fully elucidated.

Our study reveals a key oncogenic axis that connects PKA signaling, epigenetic repressors, and phosphoinositide metabolism: Activated PKA effectively stabilizes the expression level of MBD2 by recruiting the scaffold protein 14‐3‐3σ. The acquired accumulation of MBD2 then acts as a potent transcriptional repressor, targeting and binding to the promoter region of INPP5A to silence it, ultimately leading to the downregulation of INPP5A protein expression. This critical event impedes IP3 degradation, resulting in a pathological increase in intracellular IP3 levels, which in turn abnormally activates its downstream calcium signaling and oncogenic effects (such as enhancing cell proliferation, migration, and resistance to apoptosis), collectively promoting the malignant progression of pituitary tumors.

## 2 Materials and Methods

### 2.1 Human PA Samples

Sixty‐two human PA samples were collected from neurosurgical patients at Tongji Hospital (Huazhong University of Science and Technology) between September 2022 and July 2024. They were preserved via paraffin embedding, stored at −80°C, or used for primary cell extraction and cultivation. All patient‐related research was approved by the hospital's Ethics Committee.

### 2.2 Cell Lines and Primary Cell Culture

GH3, AtT20, TtT/GF, 293T, and human PA primary cells were cultured in DMEM supplemented with 10% fetal bovine serum and 1% penicillin–streptomycin. MMQ cells were maintained in RPMI 1640 supplemented with 10% fetal bovine serum and 1% penicillin–streptomycin. See Data S1 for details.

### 2.3 Tumor Xenograft Experiments

All animal studies were approved by the Animal Care Committee of Tongji Hospital (TJH‐202206015). Subcutaneous xenograft models were established as previously described. These models were grouped by treatment. Tumor‐bearing mice were monitored daily for survival; body weight and subcutaneous tumor size were measured regularly. Tumor volume was calculated as 0.5 × longest diameter × (shortest diameter) [2]. Post‐experiment, tumors were harvested and weighed. Details are in the Data S1.

### 2.4 Enzyme Linked Immunosorbent Assay (ELISA)

ELISA was performed as described previously [9]. See Data S1 for details.

### 2.5 Western Blot (WB)

WB was performed as described previously [8]. See Data S1 for details and antibodies.

### 2.6 Immunofluorescence (IF)

IF were performed as described previously [8]. See Data S1 for details.

### 2.7 Cell Proliferation Assay, Colony Formation Assay, Wound Healing Assay, Cell Invasion Assay, and 5‐Ethynyl‐2′‐Deoxyuridine (EdU) Proliferation Assay

Cell proliferation assay, colony formation assay, wound healing assay, cell invasion assay, and 5‐Ethynyl‐2′‐deoxyuridine (EdU) proliferation assay were performed as described previously. See Data S1 for details.

### 2.8 Plasmids and Transfection

The stable overexpression and short hairpin RNA (shRNA) plasmid of INPP5A and MBD2 were constructed by GeneChem Co. Ltd. The overexpression plasmids for wild‐type INPP5A (WT‐INPP5A), mutant INPP5A (MUT‐INPP5A), wild‐type MBD2 (WT‐MBD2), mutant MBD2 (MUT‐MBD2) (see Table S1) were also constructed by GeneChem Co. Ltd. A flag tag was fused to the 5′ end of all target sequences. All the above plasmids were co‐transfected into 293T cells together with packaging plasmids (psPAX2 and pMD2.G; purchased from Sangon Biotech). The shRNAs targeting MBD2 and INPP5A were constructed by Sangon Biotech (Table S2). See Data S1 for details and antibodies.

### 2.9 Real‐Time Quantitative Polymerase Chain Reaction (RT‐qPCR)

RT‐qPCR was performed as described previously. Primers used in this study are listed in Table S3. See Data S1 for details.

### 2.10 Co‐Immunoprecipitation (Co‐IP)

Cells were lysed on ice for 30 min in IP assay buffer (Servicebio) containing phosphatase and protease inhibitors, then centrifuged. Supernatants were immunoprecipitated using antibodies prebound to Protein A/G Magnetic Beads (MCE). The resulting immune complexes were separated by SDS‐PAGE and subjected to Western blotting with the indicated antibodies. Details are provided in the Data S1.

### 2.11 Mass Spectrometry (MS)

Co‐IP magnetic beads were digested with trypsin at 37°C overnight. The next day, the digested eluate was purified, vacuum‐dried, and stored at −20°C for mass spectrometry (MS) analysis. MS data were collected using a Q Exactive HF mass spectrometer. See Data S1 for further details.

### 2.12 Flow Cytometry

Cell apoptosis was assessed with an Annexin V‐FITC/PI apoptosis assay kit (Yeasen) according to the manufacturer's instructions. Details are provided in the Data S1.

### 2.13 Chip‐qPCR

ChIP‐qPCR was performed as previously described. Cells (4 × 10^7^) were collected, followed by ChIP assays with anti‐MBD2 or IgG. Primer sequences to detect the MBD2 binding site along the INPP5A promoter are listed in Table S3.

### 2.14 DNA Pull‐Down Assay

The DNA pull down assay was conducted with a DNA pull‐down kit (JKR23006A, Gene Create, Wuhan, China) according to the manufacturers' instructions. Details are provided in the Data S1.

### 2.15 Statistical Analyses

All data are presented as the mean ± standard deviation. GraphPad Prism 9.0 software was utilized for statistical analysis and graph construction, while Adobe Photoshop CC2018 was employed for image cropping. For multiple group comparisons, one‐way ANOVA followed by Tukey's post hoc test was applied, whereas an unpaired *t*‐test was used for comparisons between two groups. The data are expressed as the mean ± standard deviation. A *p*‐value < 0.05 was regarded as statistically significant (denoted as *p < 0.05, **p < 0.01, ***p < 0.001, ****p < 0.0001).

## 3 Result

### 3.1 INPP5A Is Significantly Downregulated in PitNETs and Correlates With Malignant Progression

**FIGURE 1:**
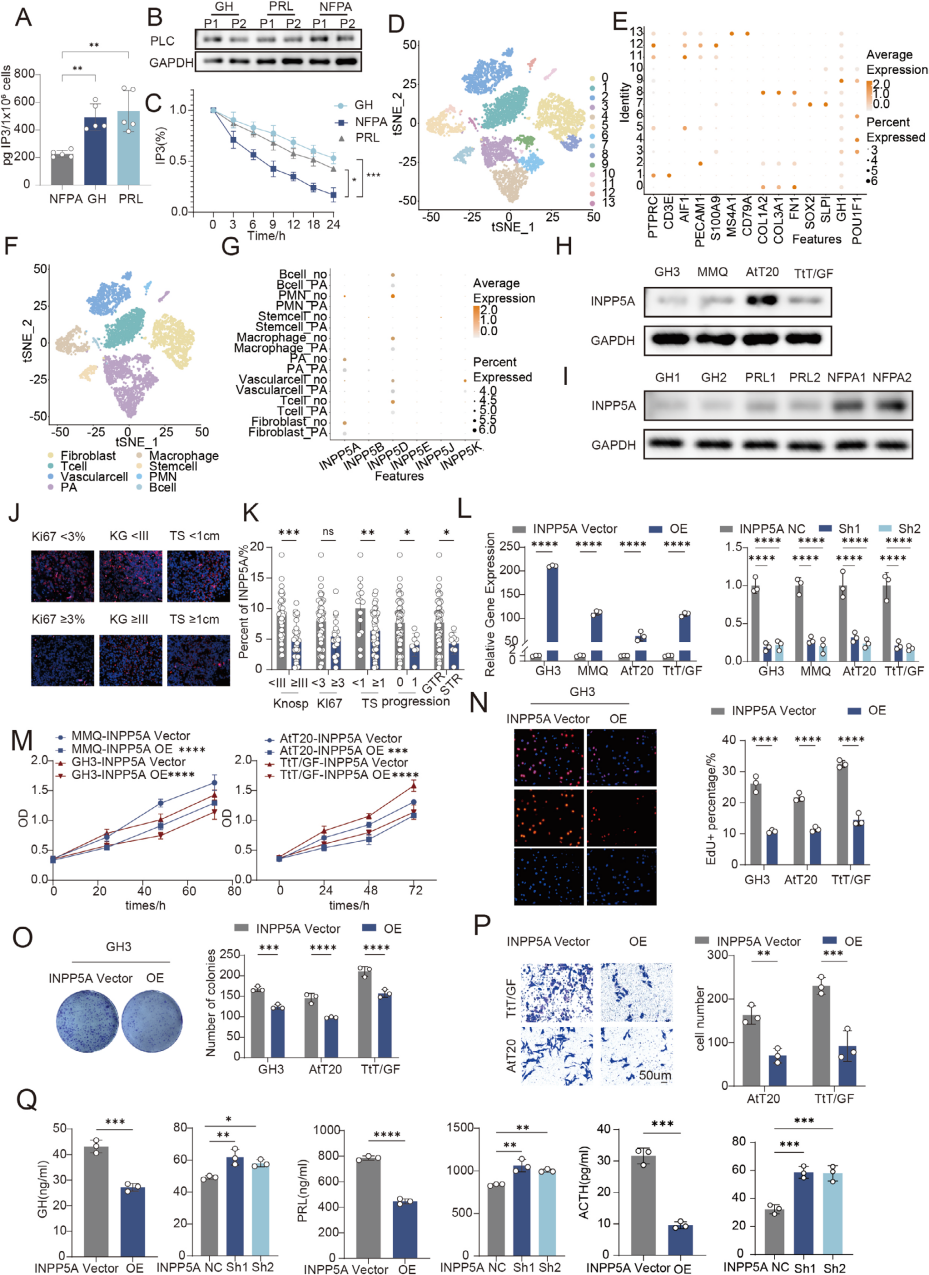
The decrease in INPP5A is associated with elevated IP3 and tumor progression in PitNET. INPP5A inhibits the proliferation, migration, and hormone secretion abilities of pituitary tumors. (A) ELISA assay of intracellular IP3 concentrations in nonfunctional pituitary adenoma (NFPA), growth hormone adenoma (GH), and prolactin adenoma (PRL) (*n* = 5). (B) Western Blot (WB) analysis of PLC expression in GH, PRL, and NFPA samples (*n* = 3). (C) Time‐course ELISA showing the decline of intracellular IP3 levels in GH, NFPA, and PRL cells relative to baseline (*n* = 5). (D) t‐SNE plot showing clustering of single‐cell RNA‐sequencing data from pituitary tumor samples. (E) Dot plot showing marker gene expression across identified cell clusters; dot size indicates the percentage of expressing cells and color intensity represents average expression. (F) tSNE plot with cell type annotations, showing cell types including Macrophages, T cells, B cells, Fibroblasts, Stem cells, Vascular cells, and Pituitary Adenoma (PA) cells. (G) Dot Plot of differential expression of INPP5 family genes between normal pituitary tissues and pituitary tumors. (H) WB analysis of INPP5A expression in PA cell lines (GH3, MMQ, AtT20, TtT/GF). (I) WB analysis of INPP5A expression in GH, PRL, and NFPA cells (*n* = 3). (J, K) Representative Immunofluorescence images of INPP5A expression in 50 pituitary tumor tissues and its relationship with Ki‐67, Knosp grade (KG), and tumor size (TS) and stained with DAPI (blue), anti‐INPP5A (red) antibodies. Scale bar, 50 μm. (L) RT–qPCR analysis of INPP5A mRNA expression in pituitary tumor cell lines following INPP5A overexpression or knockdown (*n* = 3). (M) CCK‐8 assay evaluating cell proliferation after INPP5A overexpression in GH3, MMQ, AtT20, and TtT/GF cells (*n* = 6). (N) EdU incorporation assay assessing DNA synthesis in GH3 cells after INPP5A overexpression (*n* = 3). (O) Colony formation assay evaluating long‐term proliferative capacity of GH3 cells with INPP5A overexpression (*n* = 3). (P) Transwell invasion assay assessing migratory ability of AtT20 cells following INPP5A overexpression (*n* = 3). (Q) ELISA analysis of hormone secretion, including GH (GH3), PRL (MMQ), and ACTH (AtT20), after INPP5A overexpression or knockdown (*n* = 3). Scale bar, 50 μm. Data are presented as mean ± SD. Statistical significance was determined using unpaired two‐tailed Student's *t*‐test or one‐way ANOVA with Tukey's post‐hoc test. *p < 0.05, **p < 0.01, ***p < 0.001, ****p < 0.0001.

Abnormal metabolism of IP3 plays an important role in the progression of pituitary tumors. Analysis of clinical samples revealed that functional PitNETs (growth hormone adenomas, prolactinomas) exhibit significantly elevated intracellular IP3 levels compared with nonfunctional tumors (Figure 1A), which could not be explained by differences in PLC expression (Figure 1B) but instead reflected impaired IP3 degradation (Figure 1C). Therefore, we speculate that the main reason for the elevated IP3 in functional PitNETs is reduced depletion. The metabolic enzymes responsible for IP3 degradation are mainly 5‐phosphatases and 3‐phosphatases.

Through the analysis of single‐cell sequencing samples of pituitary tumors from the GEO online database (GSE208108) (Figure 1D–F). Single‐cell transcriptomic analysis identified inositol polyphosphate 5‐phosphatase A (INPP5A) as the most markedly downregulated IP3‐metabolizing enzyme in pituitary tumor cells relative to normal pituitary tissue (Figure 1G). Consistently, INPP5A expression was lower in functional PitNET cell lines and clinical specimens (Figure 1H,I).

Importantly, reduced INPP5A expression was strongly associated with higher Ki‐67 index, increased invasiveness, and larger tumor volume, suggesting that INPP5A loss is linked to malignant progression of PitNETs (Figure 1J,K). This indicates that the decreased expression of INPP5A in pituitary tumors is associated with the malignant progression of PitNETs.

### 3.2 INPP5A Suppresses the Proliferation, Migration, and Hormone Secretion in PitNET Cells

INPP5A was overexpressed and knocked down in pituitary tumor cells (MMQ, GH3, AtT20, TtT/GF) respectively, and the transfection efficiency was verified by RT‐qPCR (Figure 1L). Subsequently, gain‐ and loss‐of‐function analyses demonstrated that INPP5A exerts a broad inhibitory effect on malignant phenotypes of PitNET cells. INPP5A overexpression consistently reduced cell proliferation (Figure 1M,N), migration (Figure 1P), and colony‐forming ability (Figure 1O), whereas INPP5A knockdown produced the opposite effects across multiple pituitary tumor cell lines (Figure S1A–E).

In hormone‐secreting PitNET models, INPP5A overexpression significantly decreased GH, PRL, and ACTH secretion, while its depletion enhanced hormone release. These findings indicate that INPP5A functions as a tumor suppressor regulating both growth and endocrine activity in PitNETs (Figure 1Q).

### 3.3 INPP5A Inhibits the Progression of PitNETs Through the PI3K/AKT Pathway

**FIGURE 2:**
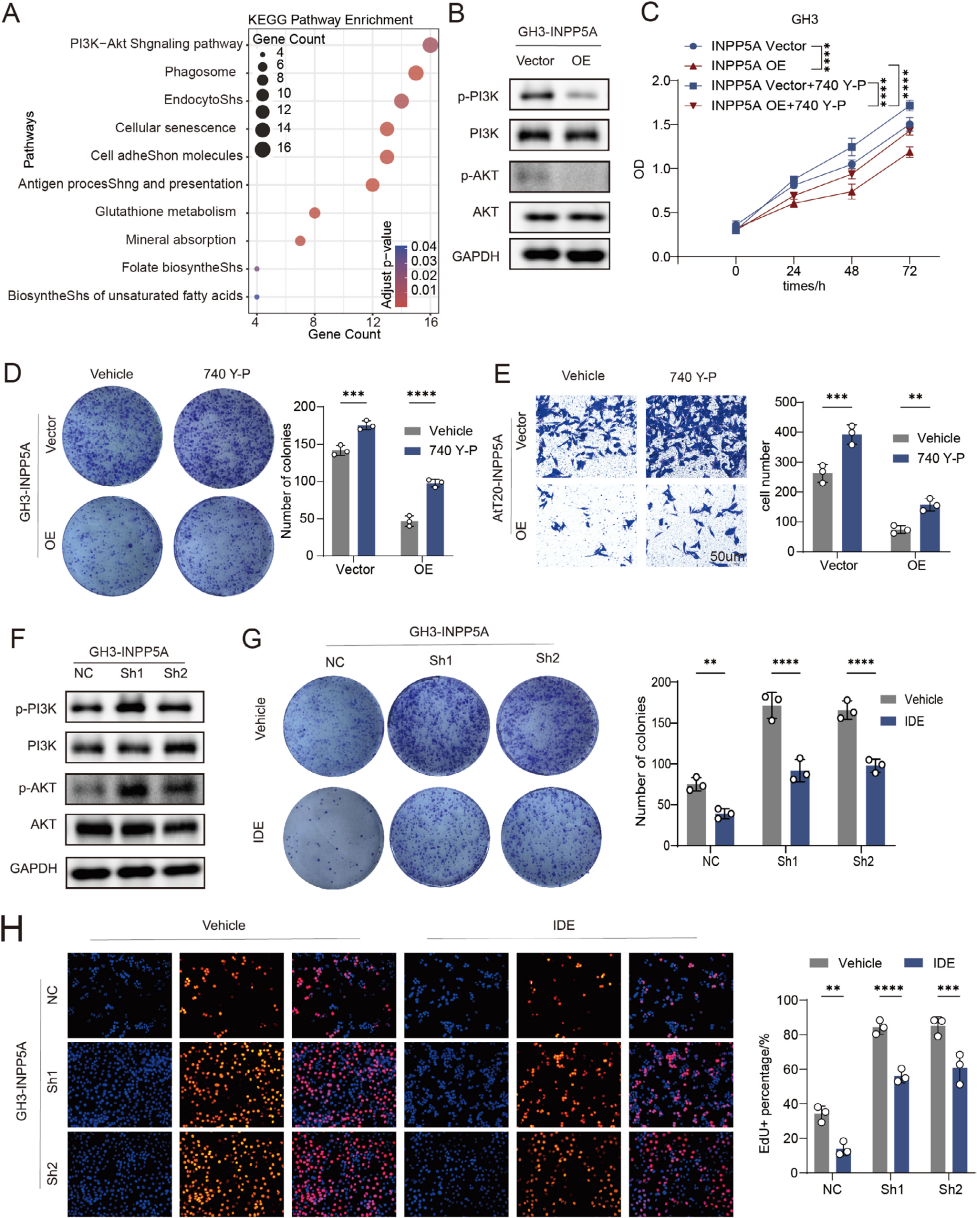
INPP5A inhibits the progression of PitNET through the PI3K/AKT pathway. (A) KEGG pathway enrichment for downregulated genes following INPP5A overexpression. (B) WB analysis of PI3K, phosphorylated PI3K (p‐PI3K), AKT, and phosphorylated AKT (p‐AKT) expression in GH3 cells with INPP5A overexpression (*n* = 3). (C) CCK‐8 assay evaluating the effect of PI3K agonist 740 Y‐P (10 μM) on GH3 cells proliferation in the presence of INPP5A overexpression (*n* = 6). (D) Colony formation of the effect of 740 Y‐P (10 μM) on GH3 cells treated with INPP5A overexpression (*n* = 3). (E) Transwell assay evaluating cell migration in AtT20 cells treated with 740 Y‐P (10 μM) and INPP5A overexpression (*n* = 3). (F) WB analysis of PI3K, p‐PI3K, AKT, and p‐AKT expression in INPP5A knock‐down GH3 cells (*n* = 3). (G) Colony formation assay assessing the PI3K inhibitor IDE (12.5 μM) on INPP5A knock‐down GH3 cells (*n* = 3). (H) EDU assays were used to assess cell proliferation of INPP5A knock‐down GH3 cells under the effect of IDE (12.5 μM) (*n* = 3). Scale bar, 50 μm. Data are presented as mean ± SD. Statistical analysis was performed using unpaired Student's *t*‐test or one‐way ANOVA with Tukey's post hoc test. **p < 0.01, ***p < 0.001, ****p < 0.0001.

Transcriptomic profiling of INPP5A‐overexpressing cells revealed significant downregulation of genes enriched in the PI3K/Akt pathway (Figure 2A), which is known to primarily affect cell proliferation and migration abilities. Consistently, INPP5A overexpression markedly reduced PI3K and Akt phosphorylation (Figure 2B), whereas INPP5A depletion enhanced pathway activation (Figure 2F). Pharmacological modulation further confirmed this relationship, as activation of PI3K signaling reversed the antitumor effects of INPP5A (Figure 2C–E, Figure S2A,B), while PI3K inhibition abrogated the pro‐tumor phenotypes induced by INPP5A knockdown (Figure 2F–H, Figure S2C,D). These data establish PI3K/Akt signaling as a critical downstream effector of INPP5A in PitNETs.

### 3.4 MBD2 Transcriptionally Represses the Expression of INPP5A

**FIGURE 3:**
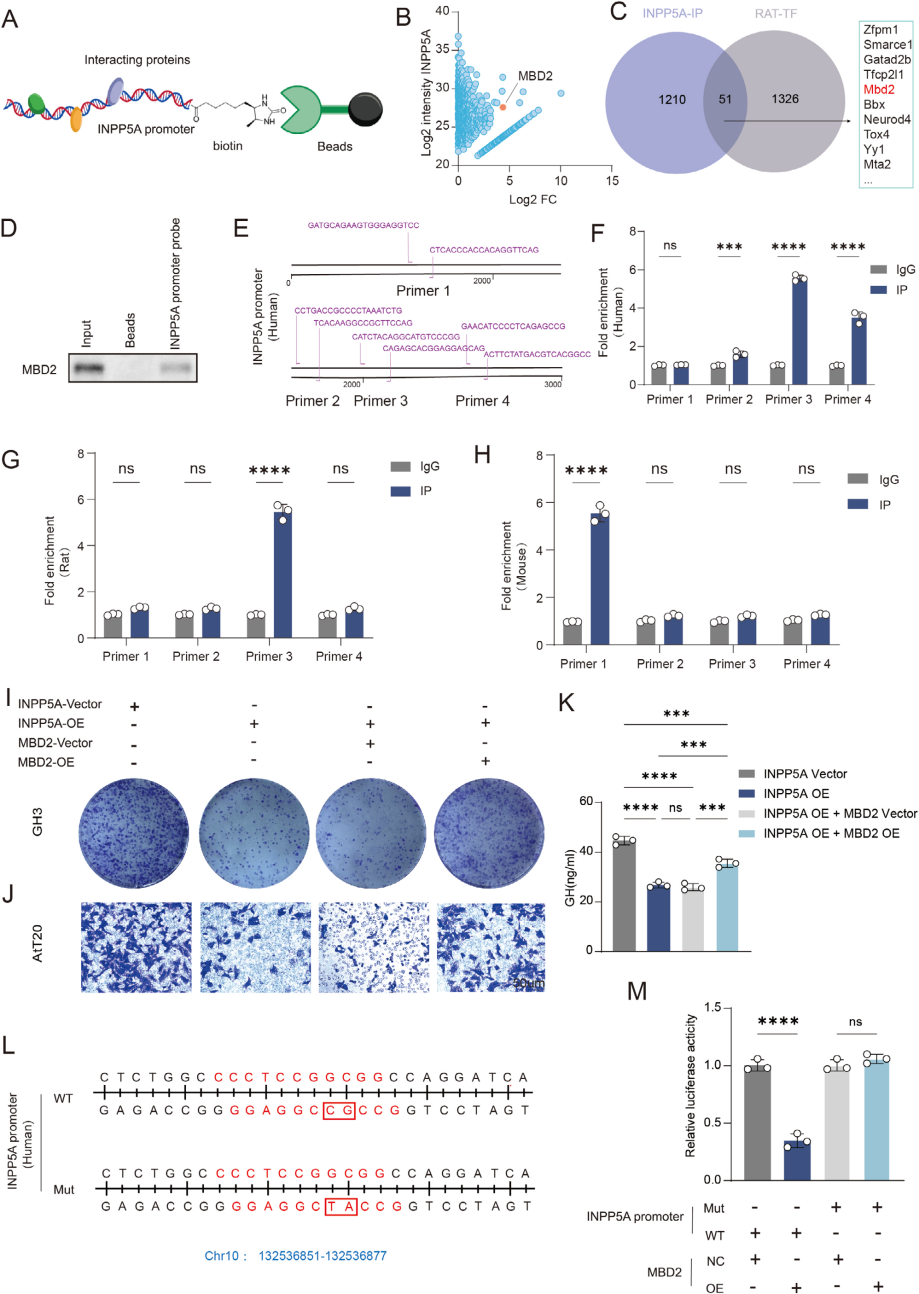
MBD2 transcriptionally inhibits INPP5A to promote pituitary tumor progression. (A) Schematic diagram of the DNA pull‐down assay using the INPP5A promoter as bait. (B) Mass spectrometry results identifying proteins bound to the INPP5A promoter in GH3 cells. (C) Venn diagram showing overlap between proteins identified by mass spectrometry and transcription factors listed in the AnimalTFDB database. (D) Western blot validation of MBD2 binding to the INPP5A promoter (*n* = 3). (E) Schematic diagram of ChIP‐qPCR primer design for the human INPP5A promoter. (F–H) ChIP‐qPCR analysis confirming MBD2 enrichment at the INPP5A promoter in human, rat, and mouse samples (*n* = 3). (I, J) Colony formation (I) and Transwell assay (J) of the effect of MBD2 overexpression on GH3 or AtT20 cells treated with INPP5A overexpression (*n* = 3). (K) ELISA analysis of GH secretion in GH3 cells with combined INPP5A and MBD2 overexpression (*n* = 3). (L) Schematic diagram showing mutation of the predicted MBD2 binding site within the INPP5A promoter. (M) Dual‐luciferase reporter assay assessing MBD2‐mediated repression of wild‐type or mutant INPP5A promoters. Scale bar, 50 μm. Data are presented as mean ± SD. Statistical analysis was performed using unpaired Student's *t*‐test or one‐way ANOVA with Tukey's post hoc test. ns, nonsignificant, ***p < 0.001, ****p < 0.0001.

To explore the reason for decreased expression of INPP5A in pituitary tumors, probes were designed for the INPP5A promoter region (Figure 3A). Through DNA pull‐down assays to detect possible bound transcriptional repressors, combined with screening against rat transcription factor databases, MBD2 was identified as a major transcriptional repressor that may bind to INPP5A to exert inhibitory effects (Figure 3B,C). The binding between the INPP5A promoter region and MBD2 was verified by WB analysis of the DNA pull‐down product (Figure 3D). Potential binding sites of MBD2 to INPP5A were predicted by integrating multiple databases via AnimalTFDB (Figure 3E, Figure S3A,B). Through ChIP‐qPCR, binding of MBD2 to the INPP5A promoter region was confirmed in three species: humans (Figure 3F), rats (Figure 3G), and mice (Figure 3H).

Rescue of the inhibitory effect of INPP5A on malignant progression of pituitary tumor cells by MBD2 was observed through colony formation assays (Figure 3I, Figure S3C,D,E), Transwell assays (Figure 3J, Figure S3F), and hormone secretion level detection (Figure 3K, Figure S3G,H). Finally, it was demonstrated by dual‐luciferase reporter assays that transcriptional expression of INPP5A is inhibited primarily through binding of MBD2 to regions 2073–2083 in humans, 850‐860 in rat, and 2825‐2835 in mouse (Figure 3L,M, Figure S3I).

### 3.5 PKA‐Mediated Phosphorylation Stabilizes MBD2 via Recruitment of 14‐3‐3σ

**FIGURE 4:**
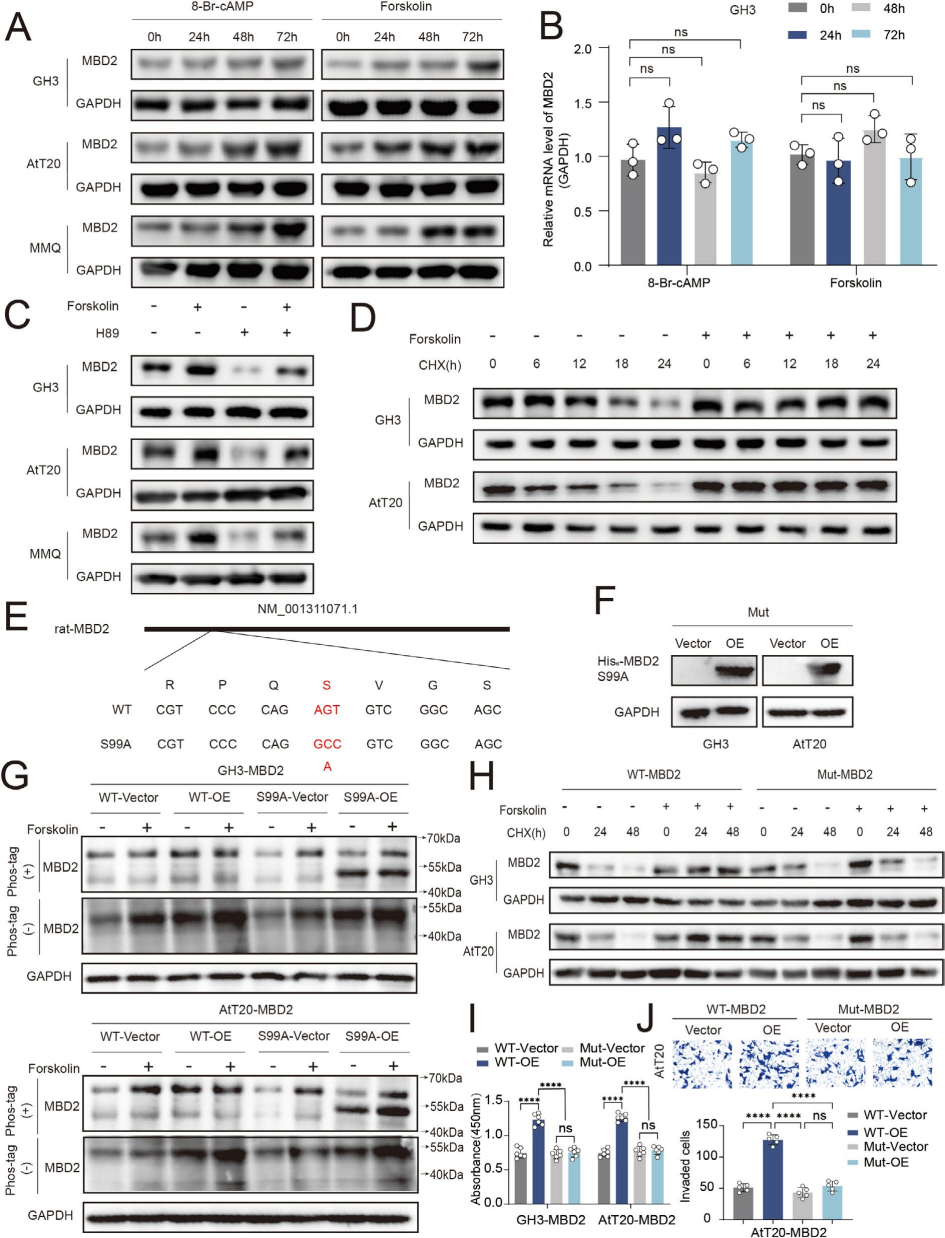
PKA binds to the S99 site of MBD2 to protect MBD2 from degradation, thereby promoting pituitary tumor progression. (A) RT–qPCR analysis of MBD2 mRNA expression in GH3 cells treated with 8‐Br‐cAMP or forskolin at indicated time points (*n* = 3). (B) WB analysis of MBD2 protein expression in GH3, AtT20 and MMQ cells following PKA activation (*n* = 3). (C) WB analysis of MBD2 expression after treatment with forskolin, the PKA inhibitor H89, or their combination (*n* = 3). (D) Cycloheximide chase assay showing MBD2 protein stability in the presence or absence of forskolin (*n* = 3). (E, F) Construction and validation of MBD2 S99A point‐mutant cell lines. (G) Representative Phos‐tag immunoblotting showing the phosphorylation status of WT and S99A MBD2 in GH3 and AtT20 cells with or without forskolin treatment (*n* = 3). (H) Cycloheximide chase assay comparing degradation rates of wild‐type and S99A‐mutant MBD2 (*n* = 3). (I) CCK‐8 assay evaluating cell proliferation in wild‐type and S99A‐mutant cells (*n* = 6). (J) Transwell invasion assay assessing migration in AtT20 cells expressing wild‐type or mutant MBD2 (*n* = 5). Scale bar, 50 μm. Data are presented as mean ± SD. Statistical analysis was performed using unpaired Student's *t*‐test or one‐way ANOVA with Tukey's post hoc test. ns, nonsignificant, ****p < 0.0001.

Activation of PKA signaling significantly increased MBD2 protein abundance without altering its mRNA levels, suggesting post‐transcriptional regulation (Figure 4A,B, Figure S4A). Conversely, inhibition of PKA activity abolished this effect (Figure 4C), and cycloheximide chase assays revealed that PKA activation markedly delayed MBD2 protein degradation (Figure 4D).

Given that PKA phosphorylates substrate proteins, it was inferred that the delayed degradation of MBD2 by PKA might be related to MBD2 phosphorylation. To identify phosphorylation sites, two databases were reviewed: NetPhos 3.1 Server and UniProt. Two high‐probability phosphorylation sites of MBD2 were identified: S99/S171/S192 in mouse (m) and S99/S169/S190 in rat (r), with S99 (m/r) as the primary residue.

Stable point mutation cell lines (S99A‐MBD2) were constructed in 293 T, GH3, and AtT20 cells (Figure 4E, Figure S4B). WB analysis was performed to verify the point mutation cell lines (Figure 4F). Representative Phos‐tag immunoblotting showing the phosphorylation status of WT and S99A MBD2 in GH3 and AtT20 cells with or without forskolin treatment. Forskolin increased the phosphorylation‐associated band shift of WT‐MBD2, whereas this effect was markedly reduced in the S99A mutant, indicating that Ser99 is an important site for MBD2 phosphorylation (Figure 4G).The effect of PKA on protecting MBD2 was significantly weakened by S99A‐MBD2, indicating faster degradation of the mutant protein (Figure 4H).

Through CCK8 (Figure 4I, Figure S4C) and Transwell assays (Figure 4J), the proliferation and migration abilities of pituitary tumor cells expressing S99A‐MBD2 were found to be significantly decreased compared to those expressing WT‐MBD2.

**FIGURE 5:**
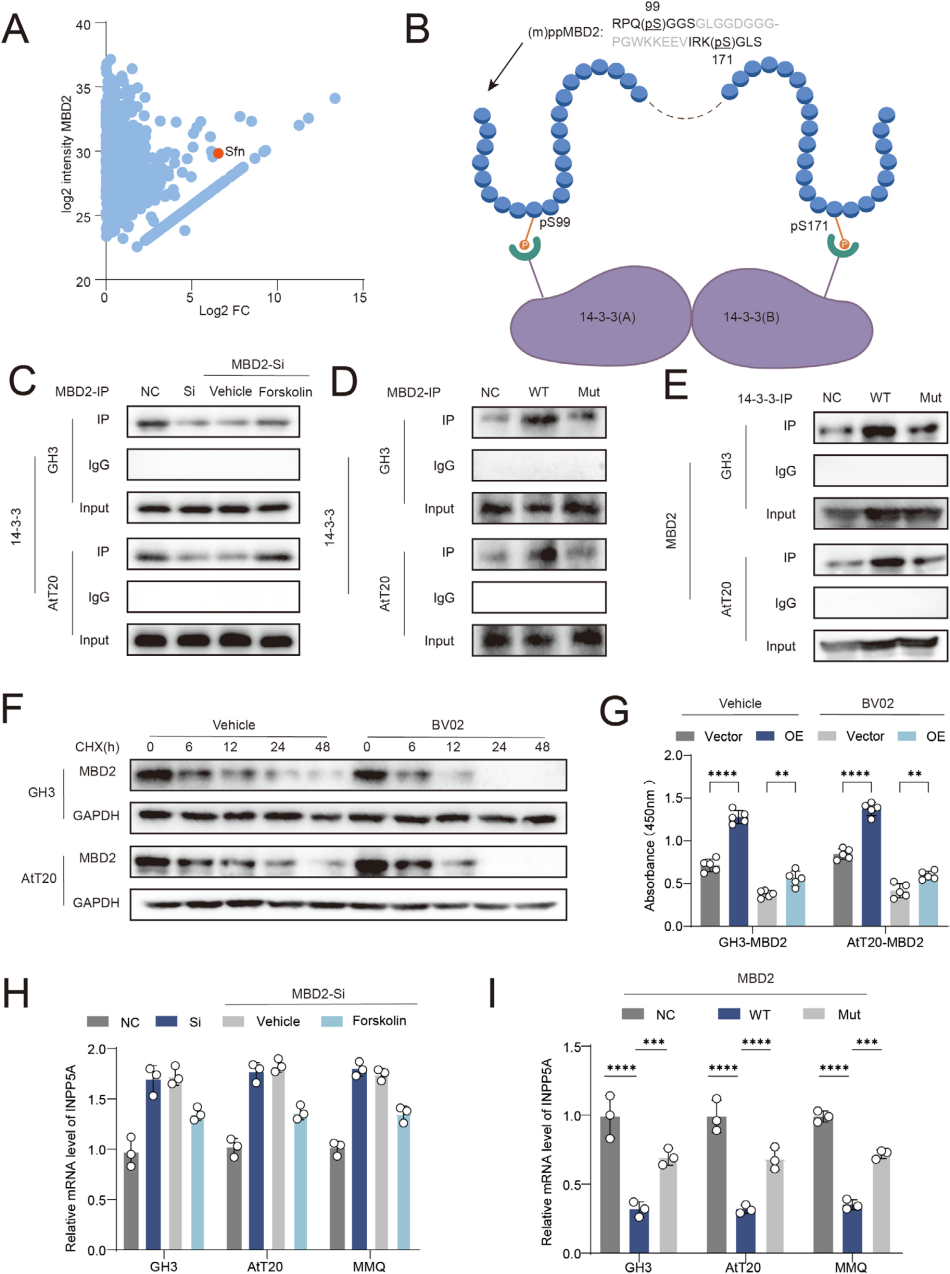
PKA phosphorylates MBD2 and then recruits 14‐3‐3 to protect MBD2 from degradation. (A) Co‐immunoprecipitation (Co‐IP) mass spectrometry results identifying MBD2‐interacting proteins. (B) Schematic illustration of phospho‐dependent interaction between MBD2 and 14‐3‐3σ. The underlined amino acid sequences indicate the consensus motif sequences recognized by 14‐3‐3σ. (C) Co‐IP confirming interaction between endogenous MBD2 and 14‐3‐3σ in GH3 and AtT20 cells (*n* = 3). (D, E) Co‐IP analysis showing loss of 14‐3‐3σ binding in S99A‐mutant MBD2 (*n* = 3). (F) Cycloheximide chase assay showing accelerated MBD2 degradation upon 14‐3‐3σ inhibition by BV02 (*n* = 3). (G) CCK‐8 assay assessing cell viability after BV02 treatment (*n* = 6). (H) RT–qPCR analysis of INPP5A expression after MBD2 knockdown with or without forskolin treatment (*n* = 3). (I) RT–qPCR analysis of INPP5A expression in cells expressing wild‐type or S99A‐mutant MBD2 (*n* = 3). Data are presented as mean ± SD. Statistical analysis was performed using unpaired Student's *t*‐test or one‐way ANOVA with Tukey's post hoc test. ***p < 0.001, ****p < 0.0001.

Mechanistically, co‐immunoprecipitation assays demonstrated that phosphorylated MBD2 interacts with the scaffold protein 14‐3‐3σ, whereas this interaction was abolished by S99 mutation (Figure 5A–E). Disruption of 14‐3‐3σ function accelerated MBD2 degradation and attenuated its oncogenic effects (Figure 5F,G). Consistently, PKA‐mediated repression of INPP5A expression was relieved by MBD2 mutation or inhibition (Figure 5H,I).

Together, these results define a PKA–MBD2–14‐3‐3σ axis that stabilizes MBD2 and sustains transcriptional repression of INPP5A in PitNET cells.

### 3.6 MBD2 Inhibitors Synergize With SSTA/DAs to Exert Anti‐Pituitary Tumor Effects

Given the critical role of MBD2 in regulating INPP5A, the efficacy differences between MBD2 inhibitors and current first‐line clinical therapies for PitNETs (somatostatin analogs, SSTAs; dopamine agonists, DAs) were compared. In the most common representative cell lines of functional PAs (GH3 and MMQ) and their primary PA cells, the effects of octreotide (OCT)/bromocriptine (BRC), the MBD2 inhibitor KCC‐07, and their combinations were evaluated.

**FIGURE 6:**
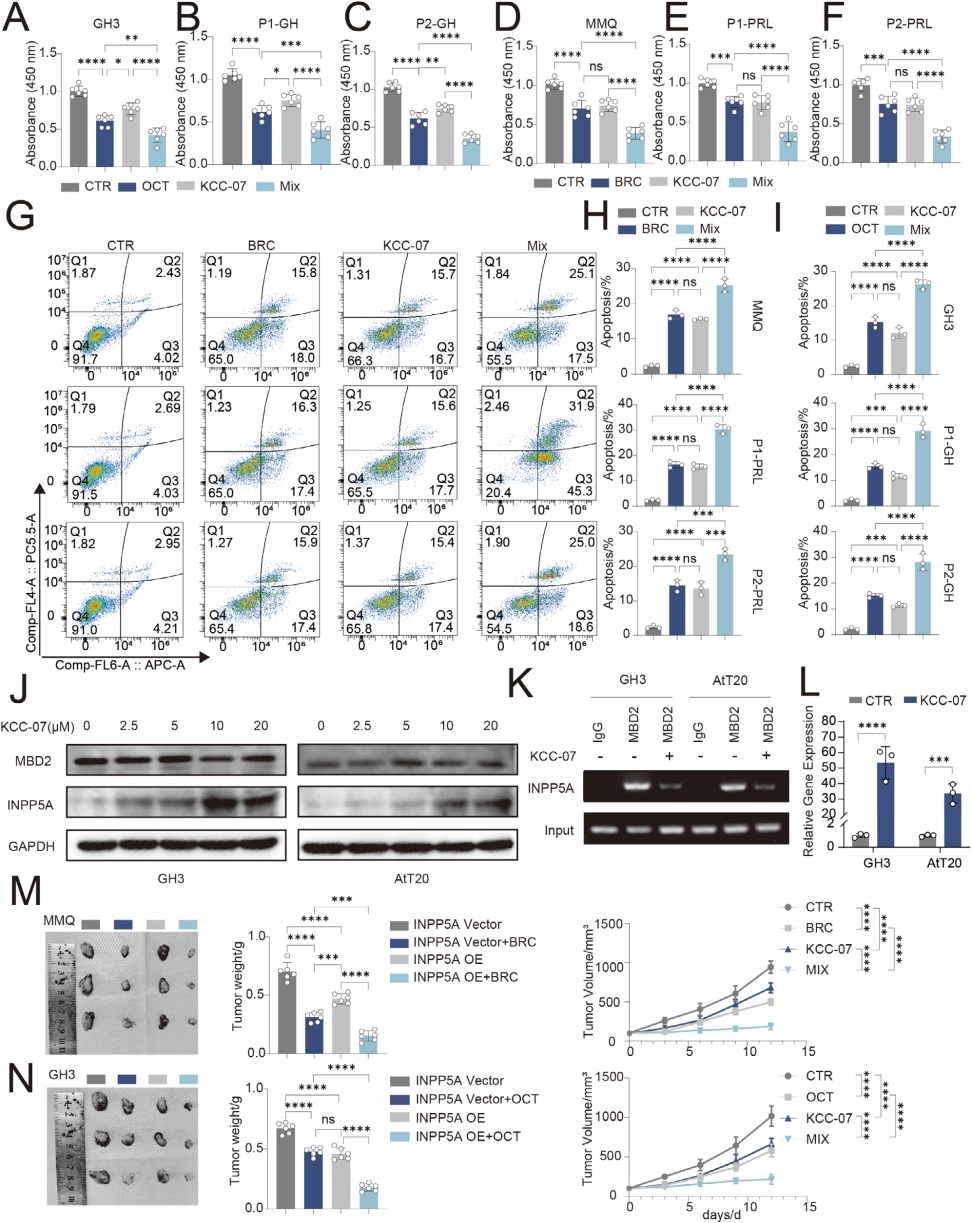
MBD2 inhibitors synergize with SSTA/DAs to exert anti‐pituitary tumor effects. (A–F) CCK‐8 assays evaluating cell viability in GH3 cells, MMQ cells, and primary GH and PRL adenoma cells treated with octreotide (OCT), bromocriptine (BRC), the MBD2 inhibitor KCC‐07, or their combinations (*n* = 6). (G–L) Flow cytometry analysis of apoptosis under the same treatment conditions (*n* = 3). (M, N) Representative images and growth curves of subcutaneous xenograft tumors derived from MMQ and GH3 cells following indicated treatments. Tumor volume was measured every 3 days. Data are presented as mean ± SD. Statistical analysis was performed using unpaired Student's *t*‐test or one‐way ANOVA with Tukey's post hoc test. *p < 0.05, **p < 0.01, ***p < 0.001, ****p < 0.0001.

Anti‐pituitary tumor proliferative effects were demonstrated by CCK‐8 assays (Figure 6A–F), with apoptosis assessed by flow cytometry (Figure 6G–L, Figure S5). These results revealed that KCC‐07 exerts antiproliferative effects, and notably, a synergistic effect was observed when KCC‐07 was combined with either OCT or BRC. These findings were robustly validated by in vivo xenograft experiments (Figure 6M,N).

## 4 Discussion

Although PitNETs are defined as benign tumors, invasive pituitary tumors have a high proliferation index and invade surrounding structures, which usually makes total surgical resection difficult and leads to a higher recurrence rate. Functional pituitary tumors can also cause abnormal hormone secretion and drug resistance, resulting in poor prognosis for patients. This requires extensive research to clarify the mechanisms of invasion and abnormal hormone secretion in invasive pituitary tumors [10, 11, 12].

In this study, we demonstrated that the expression of INPP5A is significantly inhibited in pituitary tumors and that INPP5A suppresses the proliferation, migration, and hormone secretion functions of PA cells. Previous studies have shown that INPP5A is significantly inhibited during the malignant progression of cancers. Mechanistically, we also found that INPP5A inhibits tumor growth and migration mainly by suppressing the PI3K/Akt pathway, which is a classic signaling pathway and is significantly upregulated in cancer progression [13, 14]. Moreover, the PI3K/Akt pathway also significantly promotes hormone secretion and drug resistance in pituitary tumors [15, 16]. Previous studies have found that the low expression of INPP5A in recurrent and metastatic cutaneous squamous cell carcinoma is significantly associated with poor survival [17]. The deletion of INPP5A in glioblastoma multiforme is associated with the accumulation of the phosphoinositide pathway and the progression of tumor drug resistance [5]. However, the upstream mechanisms underlying the low expression of INPP5A have not been elaborated on in these studies. From a broader tumor biology perspective, dysregulation of phosphoinositide metabolism represents a critical but often underappreciated driver of malignant progression. While aberrant activation of the PI3K/Akt pathway is a well‐established hallmark of cancer, most studies have focused on genetic or receptor‐mediated mechanisms [18, 19], whereas the contribution of upstream second messenger metabolism remains incompletely understood.

In this context, our study identifies INPP5A as a pivotal metabolic gatekeeper that constrains IP3 signaling and PI3K/Akt activation in PitNETs. Loss of INPP5A function leads to pathological accumulation of IP3, thereby amplifying oncogenic signaling cascades that promote tumor cell proliferation, migration, and hormone secretion. These findings expand the current understanding of phosphoinositide signaling in tumor biology by highlighting the functional importance of IP3 degradation, rather than production alone, in maintaining signaling homeostasis.

As a core epigenetic regulator, MBD2 mainly dynamically regulates gene transcription by recognizing DNA methylation marks. In recent years, many studies have shown that in tumors, high expression of MBD2 silences tumor suppressor genes and promotes tumor progression and metastasis [20, 21]. It has also been found in glioblastoma that MBD2 can promote tumor angiogenesis by inhibiting BAI1 [22]. Our findings extend this paradigm by demonstrating that MBD2 directly represses INPP5A transcription in PitNETs, thereby linking DNA methylation–dependent gene silencing to dysregulated phosphoinositide metabolism. Previous studies have found that PKA, as a core effector of the cAMP signaling pathway, plays a complex and multifaceted role in tumor progression [23]. Its function is influenced by subcellular localization, the type of regulatory subunits, and the tumor microenvironment, and it can promote tumor growth and metastasis [24, 25]. Aberrant activation of PKA in pituitary tumors also promotes tumor progression [26]. In previous studies conducted by our research group, it was found that PKA can promote tumor proliferation and invasion by stabilizing the expression of oncogenes [8]. In this study, we reveal that aberrant PKA activation stabilizes MBD2 through phosphorylation‐dependent recruitment of the scaffold protein 14–3‐3σ [27, 28]. This mechanism exemplifies how kinase signaling pathways can reinforce epigenetic repression by regulating the stability of chromatin‐associated proteins, providing a functional bridge between signal transduction and epigenetic control in cancer progression.

Collectively, our study delineates a PKA–MBD2–INPP5A regulatory axis that integrates kinase signaling, epigenetic repression, and phosphoinositide metabolism to drive pituitary tumorigenesis. This work not only clarifies the molecular basis of INPP5A downregulation in PitNETs but also proposes a generalizable mechanism by which dysregulated signaling pathways cooperate with epigenetic regulators to sustain oncogenic metabolic states.

From a translational perspective, the observed synergy between MBD2 inhibition and standard medical therapies suggests that targeting epigenetic–metabolic crosstalk may represent a promising strategy for overcoming therapeutic resistance in PitNETs.

## Author Contributions

Conceptualization: Y.W., Q.J., Y.H., Z.L. Methodology: Q.J., Z.W., Q.W., and S.L. Investigation: Z.Z., L.X., Z.T., K.S., T.L. and H.Z. Writing – original draft preparation: Y.W., Q.J. Writing – review and editing: Y.W., Q.J. All authors have read and agreed to the published version of the manuscript.

## Funding

This work was supported by National Natural Science Foundation of China, 82173136, 82573287.

## Disclosure

The authors have nothing to report.

## Ethics Statement

All research involving patients was reviewed and approved by the Ethics Committee of Tongji Hospital, Tongji Medical College, Huazhong University of Science and Technology (TJ‐IRB20220325). The animal research was approved by the Animal Research Ethics Committee of Tongji Medical College, Huazhong University of Science and Technology (TJH‐202206015).

## Consent

The authors have nothing to report.

## Conflicts of Interest

The authors declare no conflicts of interest.

## Supporting information


**Data S1:** Supplementary methods and materials.

**Figure S1:** (A) EdU incorporation assay assessing cell proliferation in GH3 cells following INPP5A knockdown (*n* = 3). (B) Colony formation assay evaluating long‐term proliferative capacity of GH3 cells after INPP5A knockdown (*n* = 3). (C) Transwell invasion assay of INPP5A knockdown in AtT20 cells (*n* = 3). (D) Wound‐healing assay evaluating cell migration in AtT20 and TtT/GF cells following INPP5A overexpression (*n* = 3). (E) Wound‐healing assay assessing migratory capacity of AtT20 and TtT/GF cells after INPP5A knockdown (*n* = 3). (F–H) EdU incorporation assays evaluating proliferation of AtT20 (F, G) and TtT/GF (H) cells following INPP5A overexpression or knockdown (*n* = 3). (I, J) Colony formation assays assessing long‐term proliferation of AtT20 (I) and TtT/GF (J) cells with INPP5A overexpression or knockdown (*n* = 3). Data are presented as mean ± SD. Statistical analysis was performed using unpaired Student's *t*‐test or one‐way ANOVA with Tukey's post hoc test. *p < 0.05, **p < 0.01, ***p < 0.001, ****p < 0.0001.

**Figure S2:** (A) CCK‐8 assay of the effect of 740 Y‐P (10 μM) on AtT20 (left) and MMQ (right) cells treated with INPP5A overexpression (*n* = 6). (B) Colony formation assay assessing long‐term proliferation of AtT20 cells under the same treatment conditions (*n* = 3). (C) Colony formation of the effect of IDE (12.5 μM) on shINPP5A‐AtT20 cells (*n* = 3). (D) EdU assays were used to assess cell proliferation of INPP5A knockdown AtT20 cells under the effect of IDE (12.5 μM) (*n* = 3). Data are presented as mean ± SD. Statistical analysis was performed using unpaired Student's *t*‐test or one‐way ANOVA with Tukey's post hoc test. *p < 0.05, **p < 0.01, ***p < 0.001, ****p < 0.0001.

**Figure S3:** (A, B) Schematic diagram of ChIP‐qPCR primer design for the INPP5A promoter region inrat (A) and mouse (B). (C) Colony formation assay evaluating the effect of MBD2 overexpression on GH3 cells with INPP5A overexpression (*n* = 3). (D, E) Colony formation of the effect of MBD2 overexpression on AtT20 cells treated with INPP5A overexpression (*n* = 3). (F) Transwell invasion assay evaluating migration of AtT20 cells with combined INPP5A and MBD2 overexpression (*n* = 3). (G, H) ELISA assay of PRL (G) and ACTH (H) concentration of MMQ and AtT20 cells under the effect of MBD2 overexpression on MMQ and AtT20 cells treated with INPP5A overexpression (*n* = 3). (I) Schematic diagram of mutating the MBD2 binding INPP5A promoter site in rats and mice sample identified by the AnimalTFDB database, double luciferase reporter gene experiment showed that MBD2 could not regulate mutant INPP5A promoter (*n* = 3). Data are presented as mean ± SD. Statistical analysis was performed using unpaired Student's *t*‐test or one‐way ANOVA with Tukey's post hoc test. *p < 0.05, **p < 0.01, ***p < 0.001, ****p < 0.0001.

**Figure S4:** (A) RT–qPCR of MBD2 mRNA expression in AtT20 (left) and MMQ (right) cells after administration of 8‐Br‐cAMP (50 μM) or forskolin (50 μM) at 0, 24, 48, and 72 h (*n* = 5). (B) Construction of point mutant cell lines of mice where serine at position 99 (S99A) of MBD2 is substituted with alanine in the specified cells. (C) CCK‐8 cell viability assay of wild‐type (WT) and mutant (Mut) MMQ cells (*n* = 6). Data are presented as mean ± SD. Statistical analysis was performed using unpaired Student's *t*‐test or one‐way ANOVA with Tukey's post hoc test. *p < 0.05, **p < 0.01, ***p < 0.001, ****p < 0.0001.

**Figure S5:** (A) Flow cytometry analysis of GH3 cells and GH adenoma primary cells after treatment with either OCT (200 nM), KCC‐07 (10 μM), BRC (10 μM), or Mix. Data are presented as mean ± SD. Statistical analysis was performed using unpaired Student's *t*‐test or one‐way ANOVA with Tukey's post hoc test. *p < 0.05, **p < 0.01, ***p < 0.001, ****p < 0.0001.

**Table S1:** Construction of overexpression plasmids.

**Table S2:** Construction of shINPP5A and shMBD2

**Table S3:** Primers used in this study.

**Table S4:** RNA‐Seq of INPP5A Overexpression

**Table S5:** DNA pull down MS of INPP5A

**Table S6:** MBD2 co‐ip MS

## Data Availability

The data that supports the findings of this study are available in the Supporting Information of this article.

## References

[1] L. Lu , X. Wan , Y. Xu , J. Chen , K. Shu , and T. Lei , “Classifying Pituitary Adenoma Invasiveness Based on Radiological, Surgical and Histological Features: A Retrospective Assessment of 903 Cases,” Journal of Clinical Medicine 11, no. 9 (2022): 2464, 10.3390/jcm11092464. 35566590 PMC9104472

[2] B. W. Scheithauer , K. T. Kovacs , E. R. Laws, Jr. , and R. V. Randall , “Pathology of Invasive Pituitary Tumors With Special Reference to Functional Classification,” Journal of Neurosurgery 65, no. 6 (1986): 733–744, 10.3171/jns.1986.65.6.0733. 3095506

[3] G. Hostetter , S. Y. Kim , S. Savage , et al., “Random DNA Fragmentation Allows Detection of Single‐Copy, Single‐Exon Alterations of Copy Number by Oligonucleotide Array CGH in Clinical FFPE Samples,” Nucleic Acids Research 38, no. 2 (2010): e9, 10.1093/nar/gkp881. 19875416 PMC2811007

[4] C. J. Maly , H. J. L. Cumsky , C. M. Costello , et al., “Prognostic Value of Inositol Polyphosphate‐5‐Phosphatase Expression in Recurrent and Metastatic Cutaneous Squamous Cell Carcinoma,” Journal of the American Academy of Dermatology 82, no. 4 (2020): 846–853, 10.1016/j.jaad.2019.08.027. 31437542 PMC7043906

[5] M. G. Waugh , “Chromosomal Instability and Phosphoinositide Pathway Gene Signatures in Glioblastoma Multiforme,” Molecular Neurobiology 53, no. 1 (2016): 621–630, 10.1007/s12035-014-9034-9. 25502460 PMC4703635

[6] D. Wang , J. Chen , G. Wu , et al., “MBD2 Regulates the Progression and Chemoresistance of Cholangiocarcinoma Through Interaction With WDR5,” Journal of Experimental & Clinical Cancer Research 43, no. 1 (2024): 272, 10.1186/s13046-024-03188-4. 39350229 PMC11440836

[7] K. H. Wood and Z. Zhou , “Emerging Molecular and Biological Functions of MBD2, a Reader of DNA Methylation,” Frontiers in Genetics 7 (2016): 93, 10.3389/fgene.2016.00093. 27303433 PMC4880565

[8] B. Xing , Z. Lei , Z. Wang , et al., “A Disintegrin and Metalloproteinase 22 Activates Integrin Beta1 Through Its Disintegrin Domain to Promote the Progression of Pituitary Adenoma,” Neuro‐Oncology 26, no. 1 (2024): 137–152, 10.1093/neuonc/noad148. 37555799 PMC10768997

[9] Z. Wang , Z. Lei , Q. Wang , et al., “Connexin 36 Mediated Intercellular Endoplasmic Reticulum Stress Transmission Induces SSTA Resistance in Growth Hormone Pituitary Adenoma,” International Journal of Biological Sciences 20, no. 2 (2024): 801–817, 10.7150/ijbs.86736. 38169563 PMC10758099

[10] F. Martinez‐Mendoza , S. Andonegui‐Elguera , E. Sosa‐Eroza , et al., “Spatial Transcriptomics Reveal PI3K‐AKT and Metabolic Alterations in Aggressive, Treatment‐Resistant Lactotroph Pituitary Neuroendocrine Tumors,” Acta Neuropathologica Communications 13, no. 1 (2025): 107, 10.1186/s40478-025-02025-9. 40390063 PMC12087103

[11] O. Mete and M. B. Lopes , “Overview of the 2017 WHO Classification of Pituitary Tumors,” Endocrine Pathology 28, no. 3 (2017): 228–243, 10.1007/s12022-017-9498-z. 28766057

[12] W. Su , Z. Ye , J. Liu , et al., “Single‐Cell and Spatial Transcriptome Analyses Reveal Tumor Heterogeneity and Immune Remodeling Involved in Pituitary Neuroendocrine Tumor Progression,” Nature Communications 16, no. 1 (2025): 5007, 10.1038/s41467-025-60028-5. PMC12122724 40442104

[13] J. A. Fresno Vara , E. Casado , J. de Castro , P. Cejas , C. Belda‐Iniesta , and M. Gonzalez‐Baron , “PI3K/Akt Signalling Pathway and Cancer,” Cancer Treatment Reviews 30, no. 2 (2004): 193–204, 10.1016/j.ctrv.2003.07.007. 15023437

[14] D. A. Fruman , H. Chiu , B. D. Hopkins , S. Bagrodia , L. C. Cantley , and R. T. Abraham , “The PI3K Pathway in Human Disease,” Cell 170, no. 4 (2017): 605–635, 10.1016/j.cell.2017.07.029. 28802037 PMC5726441

[15] C. Di Pasquale , E. Gentilin , S. Falletta , et al., “PI3K/Akt/mTOR Pathway Involvement in Regulating Growth Hormone Secretion in a Rat Pituitary Adenoma Cell Line,” Endocrine 60, no. 2 (2018): 308–316, 10.1007/s12020-017-1432-0. 29080043

[16] Z. Xiao , X. Yang , K. Zhang , et al., “Estrogen Receptor Alpha/Prolactin Receptor Bilateral Crosstalk Promotes Bromocriptine Resistance in Prolactinomas,” International Journal of Medical Sciences 17, no. 18 (2020): 3174–3189, 10.7150/ijms.51176. 33173437 PMC7646122

[17] H. J. L. Cumsky , C. M. Costello , N. Zhang , et al., “The Prognostic Value of Inositol Polyphosphate 5‐Phosphatase in Cutaneous Squamous Cell Carcinoma,” Journal of the American Academy of Dermatology 80, no. 3 (2019): 626–632, 10.1016/j.jaad.2018.10.018. 30359624 PMC10577667

[18] K. Okkenhaug , M. Graupera , and B. Vanhaesebroeck , “Targeting PI3K in Cancer: Impact on Tumor Cells, Their Protective Stroma, Angiogenesis, and Immunotherapy,” Cancer Discovery 6, no. 10 (2016): 1090–1105, 10.1158/2159-8290.CD-16-0716. 27655435 PMC5293166

[19] Q. Wang , P. Zhang , W. Zhang , et al., “PI3K Activation Is Enhanced by FOXM1D Binding to p110 and p85 Subunits,” Signal Transduction and Targeted Therapy 5, no. 1 (2020): 105, 10.1038/s41392-020-00218-3. 32606397 PMC7327037

[20] S. May , H. Owen , T. J. Phesse , et al., “Mbd2 Enables Tumourigenesis Within the Intestine While Preventing Tumour‐Promoting Inflammation,” Journal of Pathology 245, no. 3 (2018): 270–282, 10.1002/path.5074. 29603746 PMC6032908

[21] L. Zhang , S. Wang , G. R. Wu , et al., “MBD2 Facilitates Tumor Metastasis by Mitigating DDB2 Expression,” Cell Death and Disease 14, no. 5 (2023): 303, 10.1038/s41419-023-05804-1. 37142578 PMC10160113

[22] D. Zhu , S. B. Hunter , P. M. Vertino , and E. G. Van Meir , “Overexpression of MBD2 in Glioblastoma Maintains Epigenetic Silencing and Inhibits the Antiangiogenic Function of the Tumor Suppressor Gene BAI1,” Cancer Research 71, no. 17 (2011): 5859–5870, 10.1158/0008-5472.CAN-11-1157. 21724586 PMC3165103

[23] H. Zhang , Y. Liu , J. Liu , et al., “cAMP‐PKA/EPAC Signaling and Cancer: The Interplay in Tumor Microenvironment,” Journal of Hematology & Oncology 17, no. 1 (2024): 5, 10.1186/s13045-024-01524-x. 38233872 PMC10792844

[24] J. Tang , W. Peng , J. Ji , et al., “GPR176 Promotes Cancer Progression by Interacting With G Protein GNAS to Restrain Cell Mitophagy in Colorectal Cancer,” Advanced Science 10, no. 12 (2023): e2205627, 10.1002/advs.202205627. 36905238 PMC10131842

[25] C. Yan , Z. Yang , P. Chen , et al., “GPR65 Sensing Tumor‐Derived Lactate Induces HMGB1 Release From TAM via the cAMP/PKA/CREB Pathway to Promote Glioma Progression,” Journal of Experimental & Clinical Cancer Research : CR 43, no. 1 (2024): 105, 10.1186/s13046-024-03025-8. 38576043 PMC10993467

[26] K. Lucia , Y. Wu , J. M. Garcia , et al., “Hypoxia and the Hypoxia Inducible Factor 1alpha Activate Protein Kinase A by Repressing RII Beta Subunit Transcription,” Oncogene 39, no. 16 (2020): 3367–3380, 10.1038/s41388-020-1223-6. 32111982 PMC7160059

[27] Y. Kim , A. Shiba‐Ishii , T. Nakagawa , et al., “Stratifin Regulates Stabilization of Receptor Tyrosine Kinases via Interaction With Ubiquitin‐Specific Protease 8 in Lung Adenocarcinoma,” Oncogene 37, no. 40 (2018): 5387–5402, 10.1038/s41388-018-0342-9. 29880877

[28] H. Zhang , T. Ma , X. Wen , et al., “SIK1 Promotes Ferroptosis Resistance in Pancreatic Cancer via HDAC5‐STAT6‐SLC7A11 Axis,” Cancer Letters 623 (2025): 217726, 10.1016/j.canlet.2025.217726. 40250791

