# Supplementary material for: The PKA/MBD2 Axis Transcriptionally Represses INPP5A to Modulate PI3K/Akt Signaling and Accelerate Pituitary Tumorigenesis
Source: CNS Neurosci Ther. 2026 Mar 19;32(3):e70817. doi: 10.1002/cns.70817 (PMC13093853; doi:10.1002/cns.70817)
Supplement: Supplementary file 1 — Data S1: Supplementary methods and materials. [file CNS-32-e70817-s012.doc]

**Methods and Materials**

**Human PA Samples**

62 PA samples were collected from patients who underwent surgical procedures at the Department of Neurosurgery, Tongji Hospital of Tongji Medical College, Huazhong University of Science and Technology, during the period from September 2022 to July 2024. Subsequently, these samples were either preserved through paraffin embedding, stored at -80°C, or utilized for the extraction and cultivation of primary cells. All research involving patients was reviewed and approved by the Ethics Committee of Tongji Hospital, Tongji Medical College, Huazhong University of Science and Technology(TJ-IRB20220325)

**Cell lines and primary cell culture**

Five cell lines were used in this experiment: PitNET cell lines (GH3, MMQ, AtT20, TtT/GF) and 293T cells. The rat PitNET cell line MMQ and mouse PitNET cell line AtT20 were generously provided by Dr. Nic Savaskan of Erlangen University Hospital. GH3 and TtT/GF cell lines were purchased from the American Type Culture Collection without mycoplasma contamination (Mycoplasma Stain Assay Kit, Beyotime, cat no. C0296, China). MMQ cells were cultured in RPMI-1640 medium (G4535, ServiceBio, Wuhan, China), while GH3, TtT/GF, AtT-20 and 293T cells were cultured in DMEM/High Glucose medium (G4612, ServiceBio, Wuhan, China). All media were supplemented with 10% fetal bovine serum (FBS, A5661701, Gibco, Thermo Fisher, Waltham, MA, USA) and 1% antibiotics (G4003, ServiceBio, Wuhan, China). Cells were maintained in a humidified incubator at 37℃ with 95% air and 5% CO2.

On the first day, freshly collected pituitary tumor tissue from surgery was placed into a 50mL centrifuge tube pre-soaked in physiological saline. The tube was then placed on ice inside an insulated container, which, along with an insulation bag, was promptly transported to the laboratory and transferred to a biological safety cabinet. In a 100-mm diameter petri dish, the tumor specimen was minced into small tissue fragments (<1 mm³) using a surgical blade. These fragments were rinsed with PBS, transferred to a 15mL centrifuge tube, and centrifuged at 1,200 r/min for 4 minutes; the supernatant was subsequently discarded. Next, 4mL of 0.25% trypsin was added to the tube, which was then placed in an incubator shaker set to 37°C and 80 rpm for 30 minutes of shaking. Following this, the tube was centrifuged again at 1,200 r/min for 4 minutes, and the supernatant was removed. A 2mL aliquot of DMEM medium—supplemented with 10% fetal bovine serum, 1% streptomycin, and 1% penicillin—was added and mixed thoroughly. The resulting tissue fluid was filtered through a 40μm filter to obtain a cell suspension, which was then transferred to a 60mm diameter petri dish. Primary cells were cultured for 24 hours in an incubator maintained at 37°C with a humidified atmosphere of 95% air and 5% CO₂. After this period, the cells were rinsed with PBS and supplied with fresh medium.

**Tumor xenograft experiments**

For *in vivo* experiments, The animal research was approved by the Animal Research Ethics Committee of Tongji Medical College, Huazhong University of Science and Technology (TJH-202206015), xenograft models using GH3 and MMQ cells were established. Specifically, nude mice were randomly assigned to multiple groups, with each mouse receiving a subcutaneous injection of 1 × 10^7^ cells in the right axillary region. Tumor dimensions were measured and documented every three days post-injection. Fourteen days after cell implantation, the tumors were excised, and their weights were recorded. These tumor specimens were then fixed in formalin and embedded in paraffin for subsequent analysis. Treatment initiation occurred on the day following tumor inoculation. Each group received daily intraperitoneal injections of a specific agent: OCT (30μg/kg), KCC-07 (100mg/kg), BRC (12mg/kg), or Mix, each dissolved in 200μL of PBS. The control group was administered an equal volume of physiological saline via daily intraperitoneal injection.

**Drug treatment**

For PI3K activator treatment *in vitro,* 740 Y-P (10 μM; MCE, Shanghai, China) dissolved in PBS was added to the culture medium. For PKA activator treatment in vitro, 8-Br-cAMP (50μM; MCE, Shanghai, China) dissolved in DMSO was added to the culture medium. For adenylate cyclase activator treatment in vitro, Forskolin (50 μM; MCE, Shanghai, China) dissolved in DMSO was added to the culture medium. For protein synthesis inhibitor treatment in vitro, Cycloheximide (20 mg/mL; MCE, Shanghai, China) dissolved in DMSO was added to the culture medium. For 14-3-3 inhibitor treatment in vitro, BV02 (5 μM; MCE, Shanghai, China) dissolved in DMSO was added to the culture medium.

Daily intraperitoneal injection of OCT (30μg/kg), KCC-07 (100mg/kg), BRC (12mg/kg), or Mix in 200μL PBS was performed for nude mice in the separate treatment group. An equal volume of physiological saline was injected daily as a control.

**Enzyme Linked Immunosorbent Assay (ELISA)**

Growth hormone, Adrenocorticotropic hormone, Prolactin and IP3 concentrations were measured via ELISA Kits for GH (Elabscience, Wuhan, China), ACTH (Elabscience, Wuhan, China), PRL (Elabscience, Wuhan, China) and IP3 (Elabscience, Wuhan, China). ELISA experiments were performed according to the manufacturer's instructions. The supernatant was centrifuged before use and stored at -80°C. Ultrasonic pyrolysis was used for lytic cell suspension (1x10^6 cells/100μL PBS) to get the cell lysate, and the lysate was stored at -80°C. IP3 concentrations (pg/mL) were normalized to cell counts in each group (1x10^6 cells/100μL PBS). GH, ACTH and PRL concentrations (ng/mL) were normalized to cell counts at the beginning of each group (1x10^6 cells/2mL medium).

**Western Blot (WB)**

GH3, MMQ, TtT/GF and AtT-20 cells were lysed using RIPA lysis buffer (G2002, Servicebio, Wuhan, China) or a nuclear and cytoplasmic protein extraction kit (P0028, Shanghai, China) containing phenylmethanesulfonyl fluoride (G2008, Servicebio, Wuhan, China) and phosphatase inhibitors (G2007, Servicebio, Wuhan, China). Protein concentrations were determined using a BCA assay kit (G2026, Servicebio, Wuhan, China). Loading buffer (G2075, Ser vicebio, Wuhan, China) was added to the lysates, followed by boiling for 15 min. The protein lysates were separated by SDS-PAGE on 6% or 10% gels and transferred onto PVDF membranes (IPFL00005, Millipore, Burlington, MA, USA). The membranes were blocked with Fast Blocking Buffer (G2052, ServiceBio, Wuhan, China) at room temperature for 15 min. The membranes were incubated with primary antibodies at 4℃ for 16h. After washing, the membranes were incubated with HRP-conjugated anti-rabbit or anti-mouse antibodies (AS014, AS003, Abclonal, Wuhan, China) at room temperature for 2 h. The target proteins were visualized and imaged using an ECL chemiluminescence kit (P10060, NCM Biotech, Suzhou, China) on a GeneGnomeGRQsystem (Syngene, Cambridge, UK). Experiments were independently repeated three times.

**Immunofluorescence（IF）**

For tissue-based analyses, harvested tumor specimens were fixed in 4% paraformaldehyde solution, followed by paraffin embedding and sectioning into 5-μm-thick slices. After standard dewaxing and rehydration steps, antigen retrieval was carried out to unmask epitopes. Subsequent to sequential incubation with primary and secondary antibodies, slides intended for immunofluorescence (IF) were mounted using an antifade medium (cat. no. G1401, Servicebio) to prevent signal quenching.

Quantitative assessment of IF-positive staining for the target proteins was conducted using ImageJ software (ImageJ bundled with 64-bit Java 8; https://imagej.nih.gov/ij/). For each sample, five non-overlapping regions were selected for analysis, with results reported as either the percentage of positively stained area, mean gray value, or proportion of positive cells. All specimens were visualized and photographed using an OLYMPUS CKX5 fluorescence microscope.

**Real-time Quantitative Reverse Transcription PCR (RT-qPCR)**

Total RNA was isolated from MMQ and AtT-20 cells using the TransZol Up Plus RNA Kit (ER501-01-V2, Beijing, China). This RNA was then reverse-transcribed into cDNA utilizing the HiScript® II QRT SuperMix for qPCR (+gDNA wiper) (R223-01, Vazyme, Nanjing, China). Quantitative PCR (qPCR) was carried out with the ChamQ Blue Universal SYBR qPCR Master Mix (Q312-03, Vazyme, Nanjing, China) on an ABI QuantStudio Real-Time Real-Time PCR System (Thermo Fisher, Waltham, MA, USA). GAPDH served as the internal control, and relative expression levels were computed using the 2^ΔΔCT^ method. The primers employed in this study were obtained from Tsingke Biotechnology, with their sequences listed in Supplementary Table S2. All experiments were independently repeated three times.

**Cell Proliferation Assay**

Cells were plated in a 96-well plate at a density of 1×10^4^ cells per well. Following 24 hours of incubation, CCK-8 reagent (Cat. No. G4103, Servicebio) was added to each well, and the plate was then incubated at 37℃ for 2 hours. Cell viability was quantified by measuring the absorbance at a wavelength of 450 nm using a microplate reader (Model: Infinite F50, Tecan).

**5-Ethynyl-2’-deoxyuridine (EdU) proliferation assay**

Cell proliferation ability was assessed by Click-iT Edu-594 Cell Proliferation Assay Kit (G1603, ServiceBio, Wuhan, China) following the guidelines. GH3, TtT/GF and AtT20 cells were planted at a density of 4×10^3^ cells per well in 24-well plates. After 24 h of oridonin treatment, the cells were incubated with 10 μM EdU (ServiceBio, Wuhan, China) for two hours. Next, the cells were fixed using Immunol Staining Fix Solution (ServiceBio, Wuhan, China) for 15 min at room temperature. After washing three times with PBS containing 3% BSA, the cells were incubated for 15 min at room temperature in PBS containing 0.3% Triton X-100. The cells were then treated with Click-iT® reaction cocktails at room temperature and out of the dark for 30 min. The fluorescence images were photographed under a fluorescence microscope (Olympus, Japan) after the nuclei were stained by Hoechst 33,342 for 10 min at room temperature. Image J software was utilized to evaluate the percentage of EdU-positive cells.

**Colony formation assay**

GH3, TtT/GF and AtT20Cells were plated in 6-well plates at a density of 1000 cells per well. After 24 hours, the medium was replaced with fresh complete medium containing the respective drug for each treatment group. The cells were cultured for two weeks, with the medium refreshed once weekly. Following fixation in 4% paraformaldehyde, the colonies were visualized using crystal violet staining.

**Transwell assay**

Transwell inserts (WG3422, ServiceBio, Wuhan, China) were placed into a 24-well-plate as the upper chamber. AtT20 and TtT/GF Cells (5x10^4^) were seeded in the upper chamber with 200μl serum-free medium. 700μl complete medium was added to the lower chamber. After incubation for 48 h, cells on the bottom surface of the transwell inserts were fixed with 4% paraformaldehyde, stained with crystal violet, and counted under a microscope (Olympus).

**Wound healing assay**

TtT/GF cells were plated in 6-well plates and grown to full confluence. Vertical scratches were then created using a 200μl pipette tip. Subsequently, the cells were rinsed with PBS to eliminate any detached cells and incubated in serum-free medium for 3 days. Images of the wound areas were captured every 24 hours, and the migration distance was quantified.

**Plasmids and transfection**

The stable overexpression and short hairpin RNA (shRNA) plasmid of INPP5A and MBD2 were constructed by GeneChem Co., Ltd. The overexpression plasmids for wild-type INPP5A (WT-INPP5A), mutant INPP5A (MUT-INPP5A), wild-type MBD2 (WT-MBD2), mutant MBD2 (MUT-MBD2) (see TableS1) were also constructed by GeneChem Co., Ltd. A flag tag was fused to the 5' end of all target sequences. All the above plasmids were co-transfected into 293T cells together with packaging plasmids (psPAX2 and pMD2.G; purchased from Sangon Biotech). The shRNAs targeting MBD2 and INPP5A were constructed by Sangon Biotech (TableS2). The above transfections were performed using Lipofectamine 3000 transfection reagent (catalog number L3000015, Vazyme Biotech) strictly according to the manufacturer's instructions. Viral supernatants were collected at 48 hours and 72 hours after transfection for infecting the indicated cell lines (GH3, MMQ, AtT20, TtT/GF,293T). Stable cell lines were obtained by selection with puromycin (2-5 μg/ml; purchased from MCE). The efficiency of overexpression or knockdown was verified by RT-qPCR or WB.

**Co-Immunoprecipitation (Co-IP)**

Cells were lysed on ice for 30 minutes in IP assay buffer (cat. no. G2038, Servicebio) supplemented with phosphatase inhibitor (cat. no. G2007, Servicebio) and protease inhibitors (cat. no. G2008, Servicebio), followed by centrifugation. The resulting supernatants were subjected to immunoprecipitation using antibodies pre-bound to Protein A/G Magnetic Beads (cat. no. HY-K0202, MCE). The immune complexes were washed with PBST (1X PBS + 0.5% Tween-20), separated by SDS-PAGE, and then analyzed by WB with the specified antibodies. Isotype-matched IgG antibodies (cat. no. sc-3882/sc-2025, Santa Cruz) served as controls to rule out non-specific interactions.

**Mass spectrometry (MS)**

Beads from Co-IP samples were incubated in reaction buffer (1% SDC/100 mM Tris-HCl, pH 8.5/10 mM TCEP/40 mM CAA) at 95°C for 10 minutes to achieve protein denaturation, cysteine reduction, and alkylation. The eluted samples were diluted with an equal volume of water, and trypsin was added at a 1:50 ratio (enzyme:protein, w/w) for overnight digestion at 37°C. On the following day, trifluoroacetic acid (TFA) was used to adjust the pH to 6.0, thereby terminating the digestion process. After centrifugation at 12,000×g for 15 minutes, the peptides were purified using homemade SDB desalting columns. The eluate was vacuum-dried and stored at -20°C for mass spectrometry analysis. Mass spectrometry data were acquired using a Q Exactive HF mass spectrometer coupled with the UltiMate 3000 RSLCnano system.

**Flow cytometry**

Cell apoptosis was evaluated using an Annexin V-FITC/PI Apoptosis Assay Kit (cat. no. 40302ES, Yeasen) according to the manufacturer’s guidelines. Briefly, cells were collected, washed twice with cold PBS, and resuspended in binding buffer containing Annexin V-FITC and PI staining solution, followed by incubation for 15 minutes at room temperature in the dark. Cell apoptosis assays were conducted on a CytoFLEX flow cytometer (Beckman). The resulting data were analyzed using FlowJo v10.8 software (BD).

**Chip-qPCR**

DNA was isolated using the SimpleChIP® Plus Enzymatic Chromatin IP Kit (Magnetic Beads) (#9005, CST, Danvers, MA, USA). Initially, cells were crosslinked with 1% formaldehyde to fix proteins to DNA, after which cell lysis and chromatin digestion were performed. The digested chromatin was diluted, and each IP sample received specific antibodies that had been pre-bound to Protein A/G Magnetic Beads (HY-K0202, MCE, Shanghai, China). Samples were incubated at 4°C for 16 hours and subsequently washed using a magnetic separation rack. Chromatin was eluted from the Antibody/Protein G Magnetic Beads and subjected to reverse cross-linking. DNA was then purified utilizing spin columns. Real-time quantitative PCR was carried out with SimpleChIP® Universal qPCR Master Mix (#88989, CST, Danvers, MA, USA). IP efficiency was calculated via the percentage of input method. All experiments were independently replicated three times.

**DNA pull-down assay**

DNA pull-down kit (JKR23006A, Gene Create, Wuhan, China) was applied to complete DNA pull-down assay, according to the manufacturers′ instructions. Bio-INPP5A promoter together with its NC probe was synthesized by Wuhan GeneCreate Co., Ltd. Then, cell lysates were obtained for co-culturing with biotin probes and magnetic beads. Finally, proteins pulled down by Biotin probes were purified and western blot was applied to examine the expression of proteins.

**Statistical analyses**

All data are presented as the mean ± standard deviation. GraphPad Prism 9.0 software was utilized for statistical analysis and graph construction, while Adobe Photoshop CC2018 was employed for image cropping. For multiple group comparisons, one-way ANOVA followed by Tukey's post-hoc test was applied, whereas an unpaired t-test was used for comparisons between two groups. A p-value < 0.05 was regarded as statistically significant.
