# Supplementary figures and images for: The PKA/MBD2 Axis Transcriptionally Represses INPP5A to Modulate PI3K/Akt Signaling and Accelerate Pituitary Tumorigenesis
Source: CNS Neurosci Ther. 2026 Mar 19;32(3):e70817. doi: 10.1002/cns.70817 (PMC13093853; doi:10.1002/cns.70817)

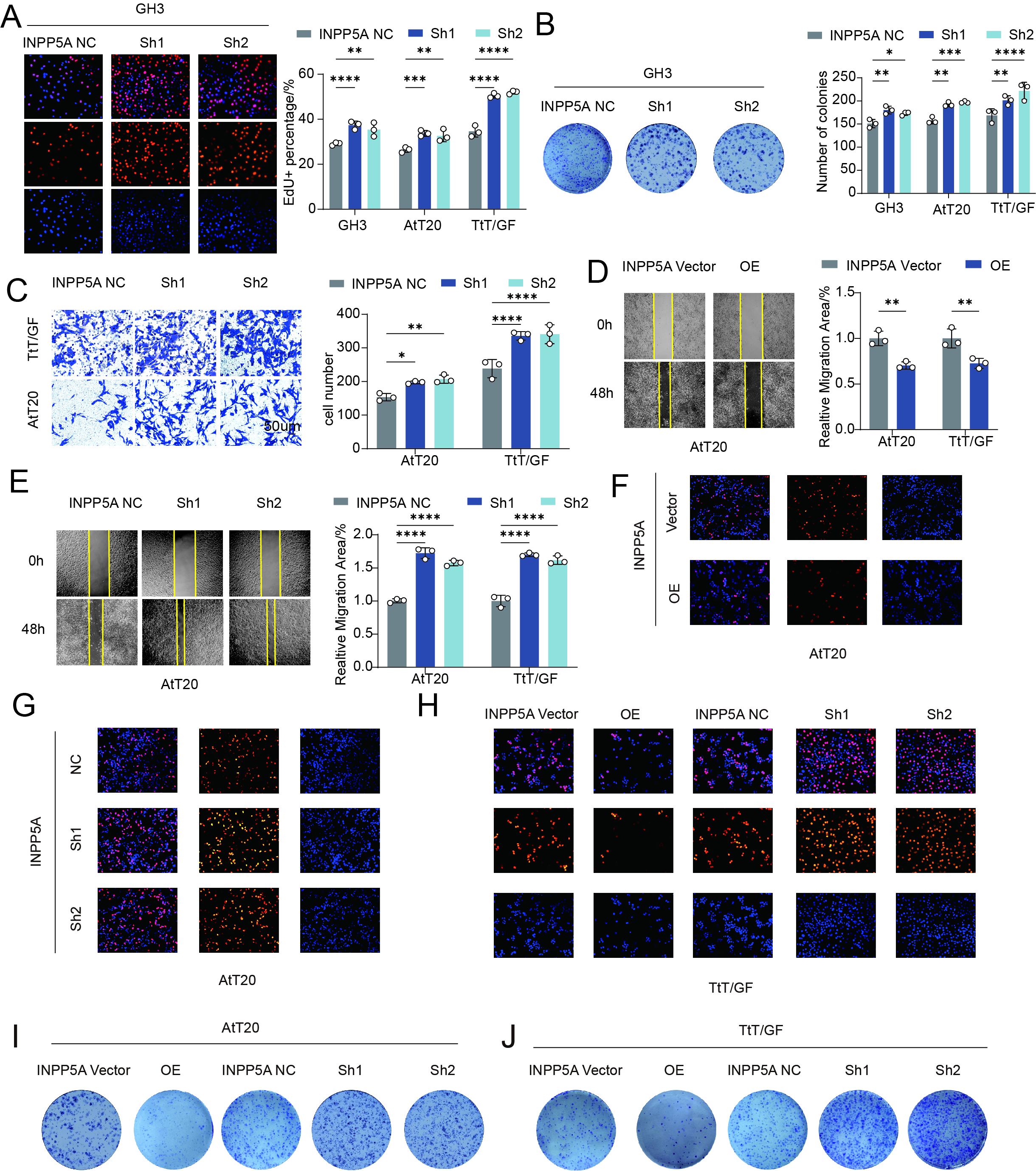

Supplement: Supplementary file 2 — Figure S1: (A) EdU incorporation assay assessing cell proliferation in GH3 cells following INPP5A knockdown (n = 3). (B) Colony formation assay evaluating long‐term proliferative capacity of GH3 cells after INPP5A knockdown (n = 3). (C) Transwell invasion assay of INPP5A knockdown in AtT20 cells (n = 3). (D) Wound‐healing assay evaluating cell migration in AtT20 and TtT/GF cells following INPP5A overexpression (n = 3). (E) Wound‐healing assay assessing migratory capacity of AtT20 and TtT/GF cells after INPP5A knockdown (n = 3). (F–H) EdU incorporation assays evaluating proliferation of AtT20 (F, G) and TtT/GF (H) cells following INPP5A overexpression or knockdown (n = 3). (I, J) Colony formation assays assessing long‐term proliferation of AtT20 (I) and TtT/GF (J) cells with INPP5A overexpression or knockdown (n = 3). Data are presented as mean ± SD. Statistical analysis was performed using unpaired Student's t‐test or one‐way ANOVA with Tukey's post hoc test. *p < 0.05, **p < 0.01, ***p < 0.001, ****p < 0.0001. [file CNS-32-e70817-s004.jpg]

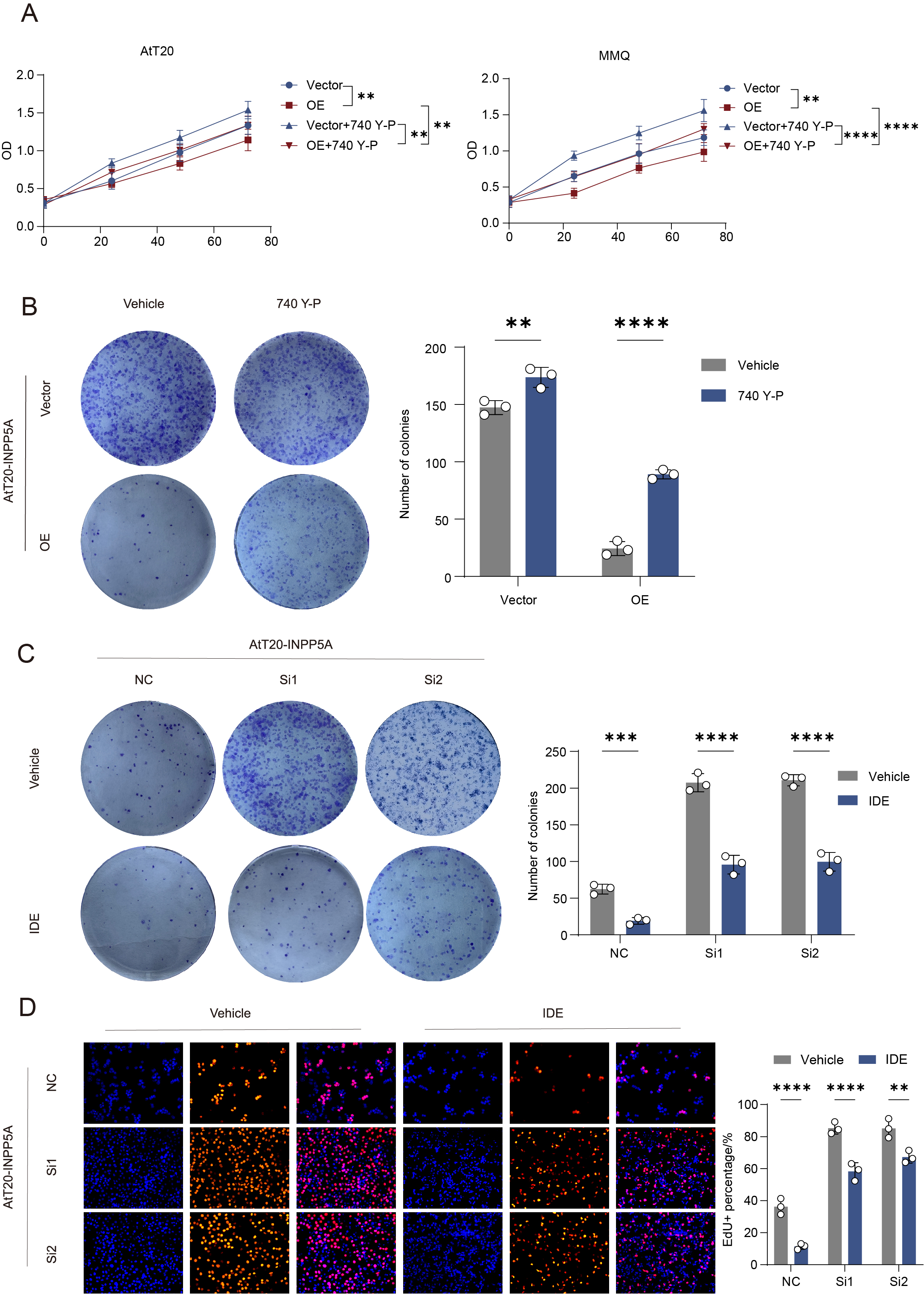

Supplement: Supplementary file 3 — Figure S2: (A) CCK‐8 assay of the effect of 740 Y‐P (10 μM) on AtT20 (left) and MMQ (right) cells treated with INPP5A overexpression (n = 6). (B) Colony formation assay assessing long‐term proliferation of AtT20 cells under the same treatment conditions (n = 3). (C) Colony formation of the effect of IDE (12.5 μM) on shINPP5A‐AtT20 cells (n = 3). (D) EdU assays were used to assess cell proliferation of INPP5A knockdown AtT20 cells under the effect of IDE (12.5 μM) (n = 3). Data are presented as mean ± SD. Statistical analysis was performed using unpaired Student's t‐test or one‐way ANOVA with Tukey's post hoc test. *p < 0.05, **p < 0.01, ***p < 0.001, ****p < 0.0001. [file CNS-32-e70817-s003.jpg]

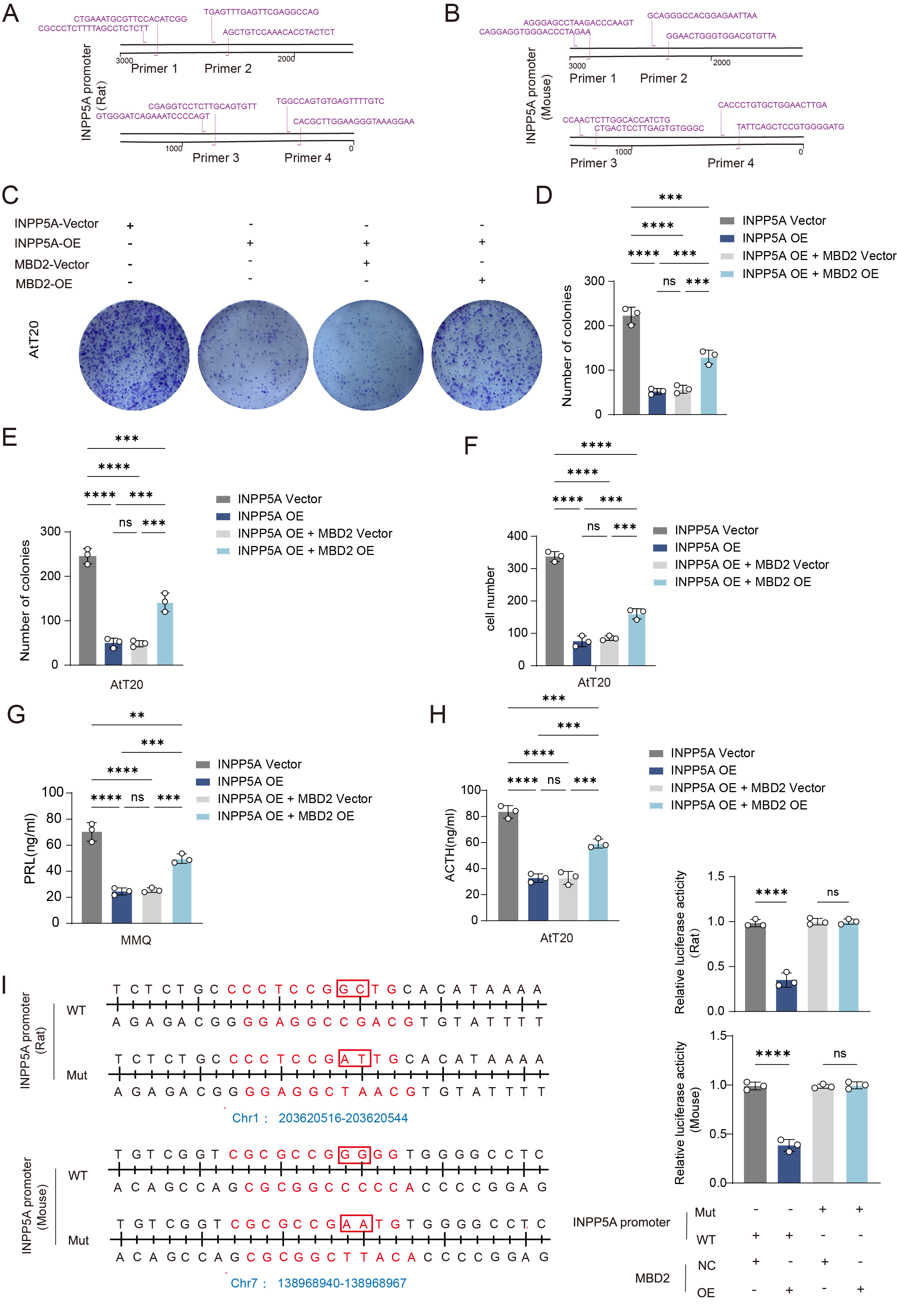

Supplement: Supplementary file 4 — Figure S3: (A, B) Schematic diagram of ChIP‐qPCR primer design for the INPP5A promoter region inrat (A) and mouse (B). (C) Colony formation assay evaluating the effect of MBD2 overexpression on GH3 cells with INPP5A overexpression (n = 3). (D, E) Colony formation of the effect of MBD2 overexpression on AtT20 cells treated with INPP5A overexpression (n = 3). (F) Transwell invasion assay evaluating migration of AtT20 cells with combined INPP5A and MBD2 overexpression (n = 3). (G, H) ELISA assay of PRL (G) and ACTH (H) concentration of MMQ and AtT20 cells under the effect of MBD2 overexpression on MMQ and AtT20 cells treated with INPP5A overexpression (n = 3). (I) Schematic diagram of mutating the MBD2 binding INPP5A promoter site in rats and mice sample identified by the AnimalTFDB database, double luciferase reporter gene experiment showed that MBD2 could not regulate mutant INPP5A promoter (n = 3). Data are presented as mean ± SD. Statistical analysis was performed using unpaired Student's t‐test or one‐way ANOVA with Tukey's post hoc test. *p < 0.05, **p < 0.01, ***p < 0.001, ****p < 0.0001. [file CNS-32-e70817-s006.jpg]

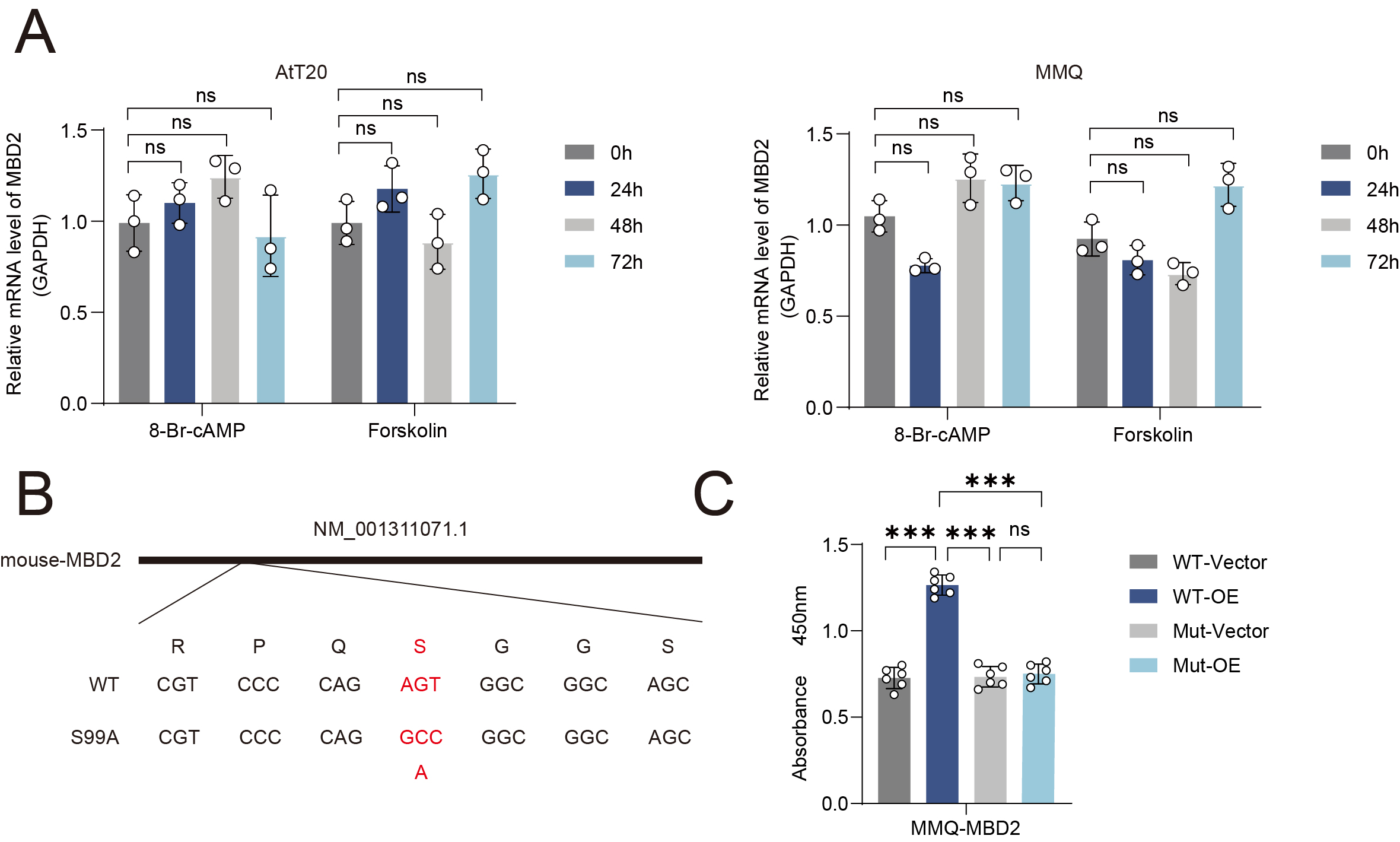

Supplement: Supplementary file 5 — Figure S4: (A) RT–qPCR of MBD2 mRNA expression in AtT20 (left) and MMQ (right) cells after administration of 8‐Br‐cAMP (50 μM) or forskolin (50 μM) at 0, 24, 48, and 72 h (n = 5). (B) Construction of point mutant cell lines of mice where serine at position 99 (S99A) of MBD2 is substituted with alanine in the specified cells. (C) CCK‐8 cell viability assay of wild‐type (WT) and mutant (Mut) MMQ cells (n = 6). Data are presented as mean ± SD. Statistical analysis was performed using unpaired Student's t‐test or one‐way ANOVA with Tukey's post hoc test. *p < 0.05, **p < 0.01, ***p < 0.001, ****p < 0.0001. [file CNS-32-e70817-s008.jpg]

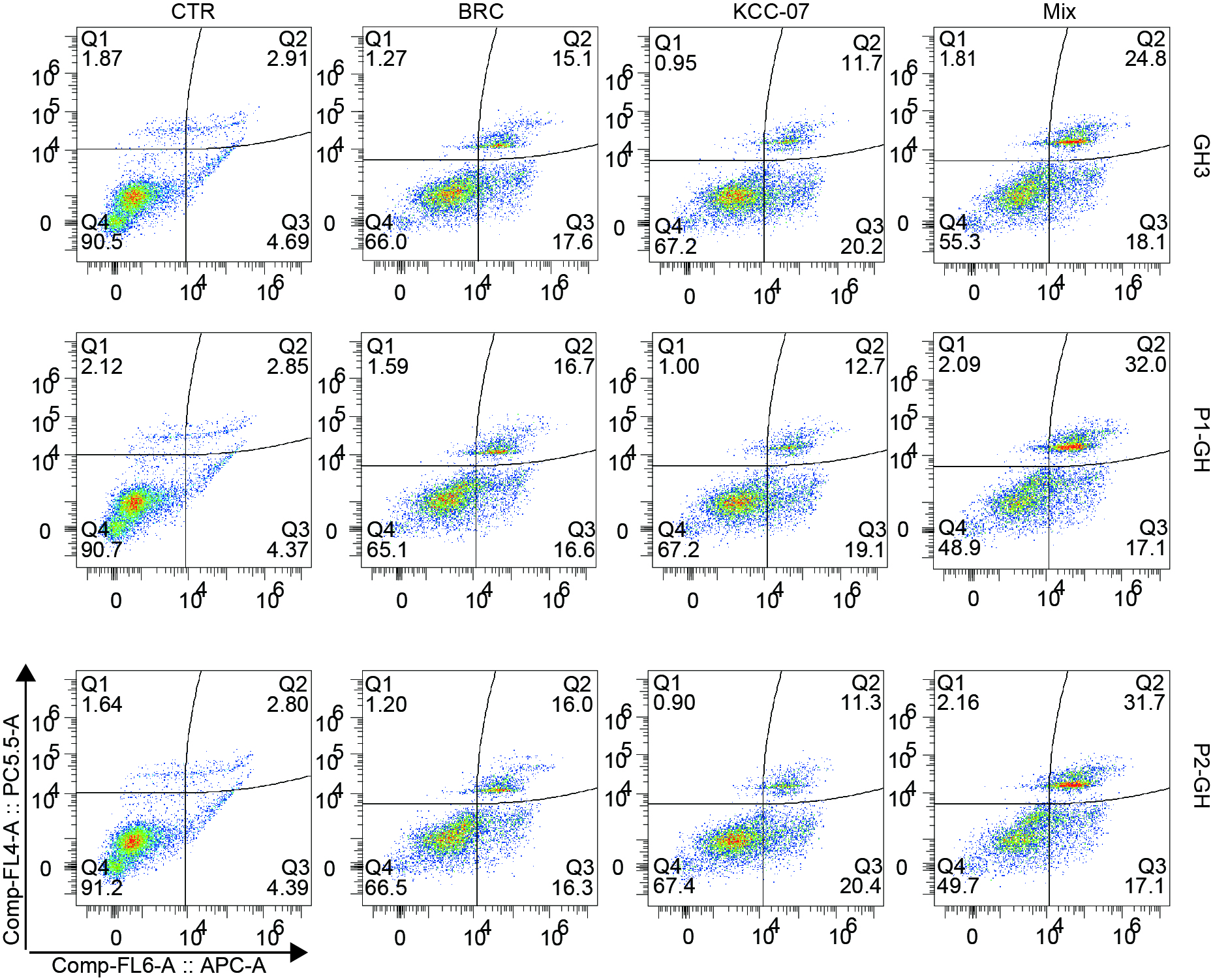

Supplement: Supplementary file 6 — Figure S5: (A) Flow cytometry analysis of GH3 cells and GH adenoma primary cells after treatment with either OCT (200 nM), KCC‐07 (10 μM), BRC (10 μM), or Mix. Data are presented as mean ± SD. Statistical analysis was performed using unpaired Student's t‐test or one‐way ANOVA with Tukey's post hoc test. *p < 0.05, **p < 0.01, ***p < 0.001, ****p < 0.0001. [file CNS-32-e70817-s011.jpg]
