# Supplementary material for: The PKA/MBD2 Axis Transcriptionally Represses INPP5A to Modulate PI3K/Akt Signaling and Accelerate Pituitary Tumorigenesis
Source: CNS Neurosci Ther. 2026 Mar 19;32(3):e70817. doi: 10.1002/cns.70817 (PMC13093853; doi:10.1002/cns.70817)
Supplement: Supplementary file 7 — Table S1: Construction of overexpression plasmids. [file CNS-32-e70817-s007.doc]

Table 1 Construction of overexpression plasmids.

| Gene ＆ Species | Transcript | cDNA | CDS Sequence | Vector |
| --- | --- | --- | --- | --- |
| INPP5A(M)  Gene ID：212111 | NM_001127363.1 | INPP5A | Full length(1239nt) | TK-PCDH-copGFP-T2A-Puro |
| INPP5A(R)  Gene ID：365382 | NM_001108923.2 | INPP5A | Full length(1239nt) |  |
| INPP5A(M)  Gene ID：212111 | NM_001127363.1 | Promoter | Promoter（3000nt） |  |
|  |  |  | c.857-858 GG > AA |  |
| INPP5A(R)  Gene ID：365382 | NM_001108923.2 | Promoter | Promoter（3000nt） |  |
|  |  |  | c.2832-2833 GC > AT |  |
| INPP5A(H)  Gene ID：3632 | NC_000010.11 | Promoter | Promoter（3000nt） |  |
|  |  |  | c.2076-2077 CG > TA |  |
| MBD2(M)  Gene ID：17191 | NM_001311071.1 | MBD2 | Full length(750nt) |  |
| MBD2(R)  Gene ID:680172 | NM_001115025.1 | MBD2 | Full length(1239nt) |  |
| MBD2(M)  Gene ID：17191 | NM_001311071.1 | MBD2 | c.295-297 AGT > GCC | TK-PCDH-copGFP-T2A-Puro-His |
| MBD2(R)  Gene ID:680172 | NM_001115025.1 | MBD2 | c.295-297 AGT > GCC |  |

Abbreviations: M, mouse; R, rat.
