# Supplementary material for: The PKA/MBD2 Axis Transcriptionally Represses INPP5A to Modulate PI3K/Akt Signaling and Accelerate Pituitary Tumorigenesis
Source: CNS Neurosci Ther. 2026 Mar 19;32(3):e70817. doi: 10.1002/cns.70817 (PMC13093853; doi:10.1002/cns.70817)
Supplement: Supplementary file 8 — Table S2: Construction of shINPP5A and shMBD2 [file CNS-32-e70817-s001.doc]

Table 2 Construction of shINPP5A and shMBD2

| Gene ＆ Species | Transcript | Position | Target Sequence (5´-3´) |
| --- | --- | --- | --- |
| INPP5A(R)  Gene ID：365382 | NM_001108923.2 | 209 | AUGUCUCAUGUGGACAAAUUU |
|  |  | 365 | AUCUACCAGUUUGACUUUAAA |
| INPP5A(M)  Gene ID：212111 | NM_001127363.1 | 370 | AUCUACCAGUUUGACUUUAAA |
|  |  | 169 | GCCUUGCACUGCCAAGAAUUU |
| MBD2(M)  Gene ID:17191 | NM_001311071.1 | 661 | CAAGAUGAUGCCUAGUAAAUU |
|  |  | 2 | UCUUUCGGAUUACUAAUAAUG |
| MBD2(R)  Gene ID: 680172 | NM_001115025.1 | 650 | CAAGAUGAUGCCUAGUAAAUU |
|  |  | 795 | ACAAACCACCCGAACAAUAAA |

Abbreviations: M, mouse; R, rat.
