# Supplementary material for: The PKA/MBD2 Axis Transcriptionally Represses INPP5A to Modulate PI3K/Akt Signaling and Accelerate Pituitary Tumorigenesis
Source: CNS Neurosci Ther. 2026 Mar 19;32(3):e70817. doi: 10.1002/cns.70817 (PMC13093853; doi:10.1002/cns.70817)
Supplement: Supplementary file 9 — Table S3: Primers used in this study. [file CNS-32-e70817-s005.doc]

Table 3 Primers used in this study.

|  | Gene ＆ Species | Forward (5´-3´) | Reverse (5´-3´) |
| --- | --- | --- | --- |
| RT-qPCR | INPP5A(M) | AGACCCCAGCCCTACCG | GTGCTCTCCAAAGTGTCCGA |
|  | INPP5A(R) | ACTTCACGGCACTAGGAAGC | GTGTACATGGGCGTGAGGTT |
|  | MBD2(M) | CCGGCTCATTAAGAAGCAGAC | TATGCAGGCAATGGAGCTGAG |
|  | MBD2(R) | CCGGCAAGATGATGCCTAGT | AGCCAAACAGCAGGGTTCTT |
|  | GAPDH(M) | AAGAGGGATGCTGCCCTTAC | TACGGCCAAATCCGTTCACA |
|  | GAPDH(R) | GCGAGATCCCGCTAACATCA | ATTCGAGAGAAGGGAGGGCT |
| CHIP-qPCR | MBD2(R) | GATGCAGAAGTGGGAGGTCC | CTCACCCACCACAGGTTCAG |
|  |  | CCTGACCGCCCCTAAATCTG | TCACAAGGCCGCTTCCAG |
|  |  | CATCTACAGGCATGTCCCGG | CAGAGCACGGAGGAGCAG |
|  |  | GAACATCCCCTCAGAGCCG | ACTTCTATGACGTCACGGCC |
|  | MBD2(M) | TATTCAGCTCCGTGGGGATG | CACCCTGTGCTGGAACTTGA |
|  |  | CTGACTCCTTGAGTGTGGGC | CCAACTCTTGGCACCATCTG |
|  |  | GGAACTGGGTGGACGTGTTA | GCAGGGCCACGGAGAATTAA |
|  |  | AGGGAGCCTAAGACCCAAGT | CAGGAGGTGGGACCCTAGAA |
|  | MBD2(R) | CACGCTTGGAAGGGTAAAGGAA | TGGCCAGTGTGAGTTTTGTC |
|  |  | CGAGGTCCTCTTGCAGTGTT | GTGGGATCAGAAATCCCCAGT |
|  |  | AGCTGTCCAAACACCTACTCT | TGAGTTTGAGTTCGAGGCCAG |
|  |  | CTGAAATGCGTTCCACATCGG | CGCCCTCTTTTAGCCTCTCTT |

Abbreviations: M, mouse; R, rat.

Primary antibodies used for the study were as follows: anti-PLCB2 (cat. no. 27456-1-AP, Proteintech), anti-GAPDH (cat. no. 10494-1-AP, Proteintech), anti-INPP5A (cat. no. 21723-1-AP, Proteintech), anti-PI3 Kinase p110 Beta (cat. no. 20584-1-AP, Proteintech), anti- Phospho-PI3 Kinase p110 beta (cat. no. BS-6417R, ThermoFisher), anti-AKT (cat. no. 10176-2-AP, Proteintech), anti-Phospho-AKT (cat. no. 28731-1-AP, Proteintech), anti-MBD2 (cat. no. 55200-1-AP, Proteintech), anti-His-tag (cat. no. 10001-0-AP, Proteintech), anti-14-3-3 (cat. no. 14503-1-AP, Proteintech)
